# Dopamine D1 receptor agonist alleviates post-weaning isolation-induced neuroinflammation and depression-like behaviors in female mice

**DOI:** 10.1186/s12993-025-00269-y

**Published:** 2025-03-10

**Authors:** Zi-Wei Zhao, Yun-Chen Wang, Pei-Chun Chen, Shun-Fen Tzeng, Po-See Chen, Yu-Min Kuo

**Affiliations:** 1 Institute of Basic Medical Sciences, College of Medicine, National Cheng Kung University, Tainan, 70101 Taiwan; 2 Department of Cell Biology and Anatomy, College of Medicine, National Cheng Kung University, Tainan, 70101 Taiwan; 3 Department of Physiology, College of Medicine, National Cheng Kung University, Tainan, 70101 Taiwan; 4 Department of Life Sciences, College of Bioscience and Biotechnology, National Cheng Kung University, Tainan, 70101 Taiwan; 5 Department of Psychiatry, College of Medicine, National Cheng Kung University Hospital, National Cheng Kung University, Tainan, 70101 Taiwan; 6 Institute of Behavioral Medicine, College of Medicine, National Cheng Kung University, Tainan, 70101 Taiwan

**Keywords:** Social isolation, Depression, Microglial activation, D1-like dopamine receptor, Medial prefrontal cortex

## Abstract

**Background:**

Major depressive disorder is a significant global cause of disability, particularly among adolescents. The dopamine system and nearby neuroinflammation, crucial for regulating mood and processing rewards, are central to the frontostriatal circuit, which is linked to depression. This study aimed to investigate the effect of post-weaning isolation (PWI) on depression in adolescent mice, with a focus on exploring the involvement of microglia and dopamine D1 receptor (D1R) in the frontostriatal circuit due to their known links with mood disorders.

**Results:**

Adolescent mice underwent 8 weeks of PWI before evaluating their depression-like behaviors and the activation status of microglia in the frontostriatal regions. Selective D1-like dopamine receptor agonist SKF-81,297 was administered into the medial prefrontal cortex (mPFC) of PWI mice to assess its antidepressant and anti-microglial activation properties. The effects of SKF-81,297 on inflammatory signaling pathways were examined in BV2 microglial cells. After 8 weeks of PWI, female mice exhibited more severe depression-like behaviors than males, with greater microglial activation in the frontostriatal regions. Microglial activation in mPFC was the most prominent among the three frontostriatal regions examined, and it was positively correlated with the severity of depression-like behaviors. Female PWI mice exhibited increased expression of dopamine D2 receptors (D2R). SKF-81,297 treatment alleviated depression-like behaviors and local microglial activation induced by PWI; however, SKF-81,297 induced these alterations in naïve mice. In vitro, SKF-81,297 decreased pro-inflammatory cytokine release and phosphorylations of JNK and ERK induced by lipopolysaccharide, while in untreated BV2 cells, SKF-81,297 elicited inflammation.

**Conclusions:**

This study highlights a sex-specific susceptibility to PWI-induced neuroinflammation and depression. While targeting the D1R shows potential in alleviating PWI-induced changes, further investigation is required to evaluate potential adverse effects under normal conditions.

**Supplementary Information:**

The online version contains supplementary material available at 10.1186/s12993-025-00269-y.

## Background

Major depressive disorder is a leading cause of global disability and lost productive years [1, 2], with adolescent onset typically occurring during late puberty at a prevalence rate of 4–5% [3, 4]. This onset is linked to significant physical and psychological changes related to neurodevelopment maturation [3, 4]. This emergence is associated with significant physical and psychological changes related to neurodevelopment maturation [5, 6]. Childhood maltreatment, including experiences like social neglect, peer rejection, and social isolation amplifies the likelihood of developing depression and anxiety during adolescence [5, 6].

The dopamine (DA) system plays important role in regulating various aspects of mental and emotional well-being, with its primary involvement in mood regulation, motivation, and the processing of rewards [7–9]. Influence of the frontostriatal (FS) circuit by intricate network of DA system [10, 11], is heavily involved in depression, as shown by functional magnetic resonance imaging studies over the past decade [7, 12–14]. Disruption of FS circuit is considered a correlate [15], a predictor [16], or even a cause [17] of depression.

A plethora of evidence has highlighted the interplay between the DA system and inflammatory responses [18]. Neuroinflammation would change the DA system, with pro-inflammatory cytokines possibly influencing DA receptor expression and function, potentially contributing to depressive symptoms [19–21]. Elevated inflammation is associated with decreased FS connectivity and increased severity of anhedonia in depressed patients [8, 22]. Chronic infusion of interferon-α disturbs DA system in the FS circuit and causes depression in nonhuman primates [23, 24]. Interestingly, DA signaling also regulates neuroinflammatory responses [25]. Stimulating DA receptors, such as the D1 receptor (D1R) and D2 receptor (D2R), inhibit the activation of microglia and astrocytes [26–28]. Furthermore, chronic inflammation has been recognized as a biological marker for major depressive disorder [29–31]. However, the underlying mechanism of crosstalk between the DA system and neuroinflammation in depression remains unclear.

This study aimed to investigate the interplay between inflammation and the DA system in the FS circuit of mice that experienced social isolation during adolescence. The neural and behavioral development of rodents is believed to parallel the stages of human development [32, 33]. Puberty in mice typically occurs between 4 and 6 weeks of age [34]. Therefore, to mimic the effects of social neglect and isolation experienced during adolescence, mouse pups were subjected to post-weaning isolation (PWI) for 8 weeks until early adulthood. It has been demonstrated that socially isolated adolescent mice can display depression-like symptoms similar to those seen in humans [5, 35–37]. Given the connection between neuroinflammation in the FS circuit and depression [38], we seek to elucidate how PWI affects depression-like behaviors and microglial activation changes in the FS circuit of adolescent mice. Furthermore, the equilibrium between D1R and D2R within the FS circuit is vital in mood regulation, while an aberrant D1R/D2R ratio is associated with the development of mood disorders [39–42]. It has been suggested that the antidepressant effect of ketamine is achieved by activating D1R in the medial prefrontal cortex (mPFC) [43]. Therefore, we also explored the effects of SKF-81,297 (SKF), a selective D1-like dopamine receptor agonist, on neuroinflammatory responses in the FS regions and depression-like behavior in mice that received PWI treatment. Additionally, BV2 microglial cells were used to explore the potential molecular mechanisms of SKF.

## Methods

### Animals

All experiments were approved by the National Cheng Kung University Institutional Animal Care and Use Committee (approval number: 110112), followed the National Institutes of Health Guide for the Care and Use of Laboratory Animals. C57BL/6 N mice of both sexes were procured from the National Cheng Kung University Laboratory Animal Center (AAALAC accredited) and housed in a controlled environment (24 ± 1 °C, 12-h light/dark cycle) with food and water ad libitum. Mice were randomly assigned to the group-housed (GH) and PWI groups. Weaning the mice at 3 weeks of age, then starting 8 weeks of PWI. PWI mice were individually housed without enrichments after weaning, while GH mice were housed with two to three same-sex cagemates.

A total of 112 mice were used in this study. The first study included 80 mice, divided into 4 groups (2 sexes × GH or PWI) with 19 mice in each group, to investigate PWI-induced depression-like behaviors. After the behavioral study, the mice were euthanized. From each group, 9 mice were allocated for western blot analyses, while the remaining 10 were used for immunohistochemistry. The second study included 36 mice, divided into 4 groups (GH or PWI × Vehicle (Veh) or SKF-81297) with 9 mice in each group, to investigate the potential of SKF in rescuing PWI-induced depression-like behaviors. The sample size was determined based on published studies [36, 44]. Details of animals used in this study were provided in Supplementary Table 1.

### Forced swimming test (FST)

The FST was used to assess depressive-like behavior and the potential antidepressant effects of various substances in mice [45]. Briefly, mice were placed in a tank (30 cm height, 15 cm diameter) filled with water (23–25 °C) 15 cm from the bottom for 6 min. Behavior was recorded during the last 4 min, and immobility time was presented as a percentage of immobility time.

### Sucrose preference test (SPT)

The SPT assessed mice response to rewards and susceptibility to anhedonia [45]. Mice were given two water bottles, one with distilled water and the other with 2% sucrose solution (Sigma-Aldrich, St. Louis, MO, USA) for 24 h. The positions of the bottles were alternated 12 h to prevent position preference. Sucrose preference was defined as the ratio of the consumed sucrose solution to the total consumed solution over 24 h.

### Estrus cycle determination

Estrus cycle was determined using vaginal smears as described [46, 47]. Vaginal cell samples were collected by gently flushing the vaginal cavity with 20 µl of saline and spreading them on a glass slide. Once fully air-dried, the slide was subjected to staining with Hematoxylin and Eosin (Abcam, Cambridge, UK). Vaginal secretions comprise three primary cell types: leukocytes, cornified epithelial cells, and nucleated epithelial cells. Determining the estrous cycle phase under a microscope relied on the relative proportions of these cell types in the vaginal secretions. Mice in the proestrus and estrus phases are considered sexually receptive (SR), whereas mice in diestrus and metestrus are non-receptive (NR). The estrous cycle phase was determined before conducting the FST.

### SKF administration

The SKF dosage and the timing of the post-administration behavioral test adhered to a previous publication [43]. Briefly, SKF (Cayman Chemical, Ann Arbor, MI, USA, #15067) was dissolved in artificial cerebrospinal fluid to make a 0.2% solution. One day before the 8-week PWI concluded, mice were anesthetized intraperitoneally with a mixture of 50 mg/kg Zoletil 50 (Virbac, Carros, France) and 2.5 mg/kg xylazine (Bayer, Leverkusen, Germany) and received bilateral injections of SKF (0.5 µL/side, infusion rate: 0.1 µL/min) into the prelimbic area of the mPFC (anteroposterior: +1.9 mm; mediolateral: ±0.5 mm; ventral: 2.5 mm from the bregma) using a 30-gauge stainless steel needle attached to a microsyringe that was powered by a controllable electric pump (Model: KDS 210, KD Scientific, Holliston, MA, USA). Initially, we performed stereotaxic surgery to inject Evans blue into the targeted area (stereotaxic coordinates: anteroposterior: +1.9 mm, mediolateral: ±0.5 mm, and ventral: -2.5 mm from the bregma) to confirm accurate injection placement based on a brain atlas. In the formal experiment, we verified the injection site location by assessing microglial activation (using Iba1 immunohistochemistry) along the needle injection tract one day post-surgery (Fig. 3B). To ensure their well-being, mice were kept warm until fully recovered. Following a previously published protocol [43], the FST was conducted 18 h post-SKF administration, and mice were sacrificed 6 h later.

**Fig. 1:**
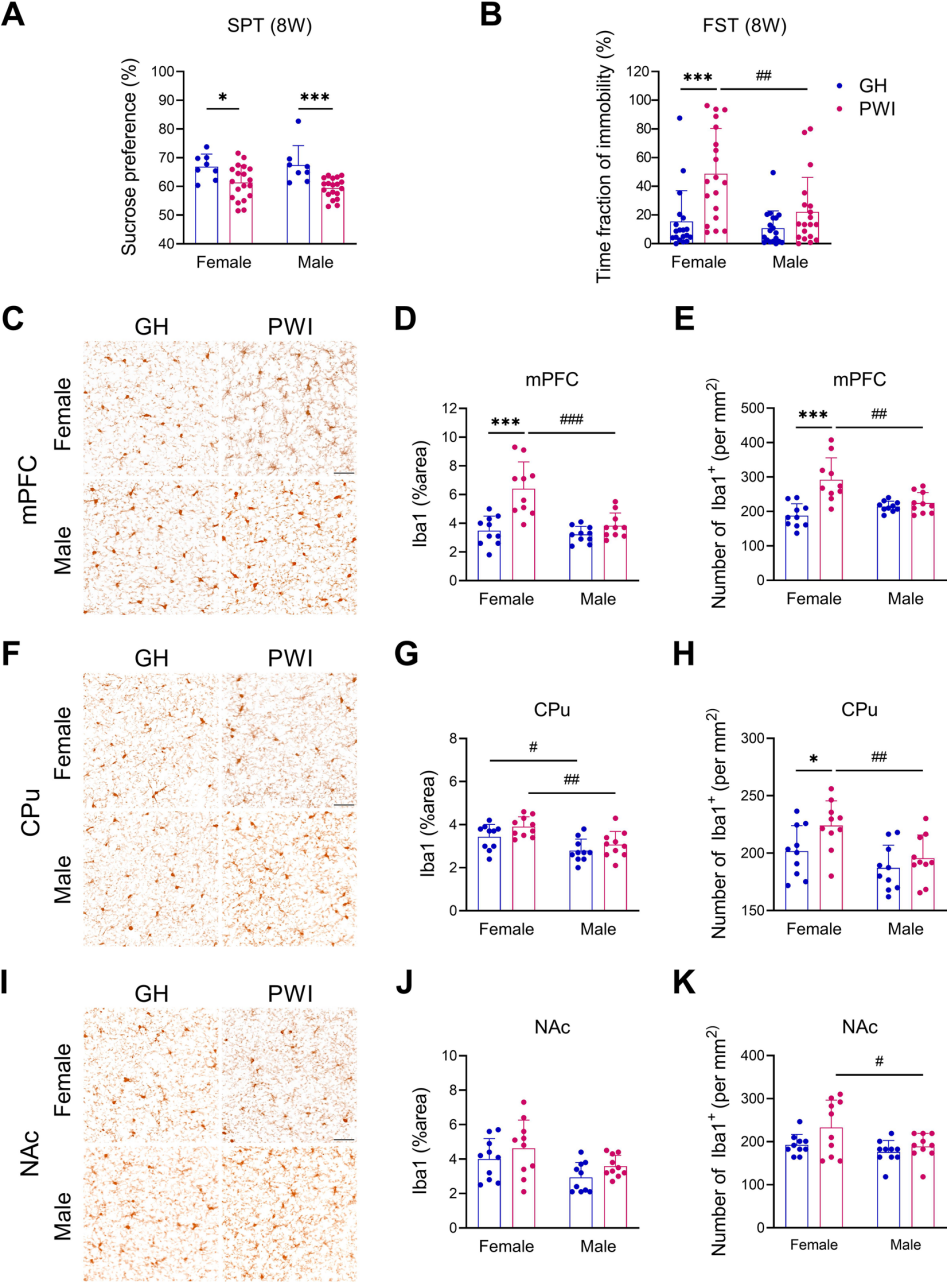
Eight-week PWI induced depression-like behaviors and microglial activation in FS circuit-related brain regions of mice. (**A**) Results of sucrose preference in SPT after 8 weeks of PWI. *n* = 8 cages of GH and 19 cages of PWI. (**B**) Results of the exhibition of immobility in FST after 8 weeks of PWI. *n* = 19 mice. (**C**, **F**, and **I**) Representative micrographs of Iba1 immunostaining in the prelimbic region of the mPFC, the middle region of the CPu, and the core region of the NA inferior to the anterior commissure. (**D**, **G**, and **J**) Quantitative results of Iba1^+^ area in mPFC, CPu, and NAc. (**E**, **H**, and **K**) Quantitative results of Iba1^+^ density in mPFC, CPu, and NAc. Data were expressed as mean ± SD. Sidak’s multiple comparisons test was used following two-way ANOVA. Post-hoc results showed that ^*^p < 0.05, ^***^p < 0.001 indicated significant differences between GH and PWI mice, while ^#^*p* < 0.05, ^##^*p* < 0.01, ^###^*p* < 0.001 represented significant differences between female and male mice

### BV2 microglial cell cultures

BV2 microglial cells (RRID: CVCL_0182) were cultured in Dulbecco’s Modified Eagle Medium F12 (DMEM/F12; Gibco, Invitrogen, Carlsbad, CA, USA) supplemented with 10% fetal bovine serum (Merck-Millipore, Billerica, MA, USA) and 1% penicillin-streptomycin (Thermo Fisher Scientific, Waltham, MA, USA). Cultures were maintained in a humidified atmosphere with 5% CO_2_ and 95% air at 37 °C. Subcultures were performed when the cell density reached 80% confluence, approximately every 2–3 days.

For experiments, BV2 cells were seeded in 12-well plates at a density of 10^5^ cells per well and cultured in 1 ml of DMEM/F12. Sixteen hours after seeding, cells were pre-treated with 2.5 µM SKF or an equal volume of 0.1% DMSO (Veh control) for 30 min. Subsequently, cells were treated with 50 ng/ml lipopolysaccharide (LPS; Escherichia coli O55:B5, Sigma-Aldrich, #L2880), 20 ng/ml recombinant mouse tumor necrosis factor (TNF; BD Biosciences, San Jose, CA, USA, #554589), or an equal volume of phosphate buffered saline (PBS, Veh control).

To assess the effects of SKF on LPS and TNF-induced inflammatory responses, a 2 (with or without SKF) x2 (with or without LPS/ TNF) experimental design was employed. Cells were collected 60 min after LPS treatment (15 min–2 h after TNF treatment), and conditioned media were collected 24 h after LPS or TNF treatment. Each analysis was based on three biological replicates obtained from three independent experiments.

### Cell viability

BV2 microglial cells were seeded in 96-well culture plates at a density of 10^4^ cells per well and incubated in 100 µl of DMEM/F12 medium until they adhered to the well bottom for 24 h. After cell adherence, 10 µl of CCK-8 reagent (TargetMol, Washington, DC, USA) was added to each well for even distribution. The cells were incubated with CCK-8 for 1 h, followed by measuring absorbance at 450 nm wavelength. This measurement allows quantification of formazan production, directly proportional to the number of living cells within each well.

### Western blot

The method for Western blotting was described elsewhere [45]. Briefly, BV2 microglial cells lysates were centrifuged at 12,000×g for 15 min at 4 °C, and protein concentrations were determined. Ten µg of proteins were loaded into each well of a polyacrylamide gel, resolved, and transferred onto a polyvinylidene fluoride membrane (Merck-Millipore, Billerica, MA, USA). The membrane was blocked with 5% non-fat milk and probed with primary antibodies diluted 1000-fold. All primary antibodies were purchased from Cell Signaling Technology (Beverly, MA, USA), except for those targeting D1R (Atlas Antibodies, Stockholm, Sweden) and D2R (Merck-Millipore). Secondary antibodies conjugated to horseradish peroxidase were added, and protein signals were detected using an ECL substrate (Perkin Elmer, Waltham, MA, USA). Membranes were imaged using X-ray films (Fujifilm, Tokyo, Japan). Supplementary Table 2 contains all the detailed information regarding the antibodies.

### Immunohistochemistry

The method for immunohistochemistry was described previously [45]. Briefly, after receiving intraperitoneally injection with a mixture of Zoletil 50 (50 mg/kg) and xylazine (2.5 mg/kg), mice were transcardially perfused with PBS and their brains were removed. The left hemispheres were post-fixed in 4% paraformaldehyde for 48 h at 4 °C, followed by 30% sucrose solution. The brains were sliced into 25 μm coronal sections and stored at -20 °C. Brain sections were treated with 5% H_2_O_2_, and blocked with 3% normal goat serum for 1 h at room temperature. The free-floating sections were probed with rabbit anti-Iba1 antibodies (1:1000; Wako Pure Chemical Industries, Osaka, Japan) overnight at room temperature followed by incubation with horseradish peroxidase conjugated goat anti-rabbit IgG (1:1000; Jackson ImmunoResearch, West Grove, PA, USA, #111-035-003) using 3,3’-diaminobenzidine tetrahydrochloride (Sigma-Aldrich) as the substrate. Supplementary Table 2 contains all the detailed information regarding the antibodies. Sections were mounted on silane-coated slides (Microslides, MUTO Pure Chemicals, Tokyo, Japan, #5116), air-dried overnight, dehydrated with escalating concentrations of ethanol, and coverslipped with Micromount xylene-based mounting medium (Leica Biosystems, Nussloch, Germany).

### Quantifying the immunostained area and cell density

We measured the areas and densities of Iba1-immunostained (Iba1^+^) cells in specific brain regions: the mPFC (anterior cingulate, prelimbic, and infralimbic subregions) at bregma 1.8 mm, anterior caudate/putamen (CPu) at bregma 0.8 mm, middle CPu at bregma 0.0 mm, posterior CPu at bregma − 0.8 mm, and nucleus accumbens (NAc) core at bregma 1.3 mm. The total Iba1^+^ signal areas and cell counts were quantified within a designated 330 μm x 330 μm box at the center of each region. Average areas and cell counts were determined for each subregion of the mPFC, CPu, and NAc core. For the SKF study, measurements were made in the prelimbic region adjacent to the injection site. Images of sections were taken by the digital camera (Model: Axiocam 305 Color, Carl Zeiss, Oberkochen, Germany) under an optical microscope (Carl Zeiss). Iba1^+^ areas were calculated using ImageJ software (version 1.51 w, NIH, Bethesda, MD, USA) by measuring areas exceeding a predefined background threshold, consistent across all sections.

### RNA extraction and Real-time PCR (RT-PCR)

Extract total RNA from BV2 microglial cells was extracted using the Quick-RNA Miniprep Plus Kit (Zymo Research, Irvine, USA, #R1057) following the manufacturer’s instructions. Treat RNA with DNase I to remove genomic DNA contamination. Reverse-transcribe purified mRNA into cDNA using the PrimeScript RT Reagent Kit (Takara, Japan, #RR037A) according to the manufacturer’s instructions. RT-PCR was performed with SYBRGreen MasterMix (Applied Biosystems, Foster City, CA, USA) in a final volume of 20 µL to quantify relative gene expression on a StepOnePlus RT- PCR system (Applied Biosystems). Each RT-PCR reaction included a subsequent melting-curve analysis to ensure specificity. The RT-PCR program consisted of an initial denaturation at 95 °C for 10 min, followed by 40 cycles of 95 °C for 15 s and 60 °C for 1 min. The primer sequences used were as follows: D1R (forward): 5′-GTAGCCATTATGATCGTCAC-3′, D1R (reverse): 5′-GATCACAGACAGTGTCTTCAG-3′; D2R (forward): 5′-CTGGTGGCCACACTGGTTAT-3′D2R (reverse): 5′-GGCACACAGGTTCAAGATGC-3′; D5R (forward): 5′-CTACGAGCGCAAGATGACC-3′, D5R (reverse): 5′-CTCTGAGCATGCTCAGCTG-3′; GAPDH (forward): 5′-TGCAGTGGCAAAGTGGAGATT-3′, GAPDH (reverse): 5′-TTGAATTTGCCGTGAGTGGA-3. Relative gene expression was quantified using cycle threshold values, with GAPDH as the internal control.

### Cytokine concentrations measurement

The concentrations of TNF and IL-6 in the conditioned media were determined by commercial mouse TNF (BD Biosciences) and IL-6 (BD Biosciences) ELISA kits following the manufacturer’s instructions.

### Statistical analysis

Data were expressed as mean ± standard deviation (SD). Significance was set at *p* < 0.05. The data followed a normal distribution. The unpaired two-tailed Student’s t-test was utilized to examine a single independent variable. To analyze the effect of a single variable in datasets with more than two groups, Dunnett’s multiple comparison tests were employed following the one-way ANOVA. Two-way ANOVA was used to analyze all 2 × 2 factorial designs (e.g., PWI×sex, PWI×SKF, LPS/TNF×SKF, and durarion×SKF) and possible interactions. Sidak’s multiple comparisons test was done if interaction or the main variables were significant. The association between FST and Iba1 signals was evaluated using the Pearson correlation. All statistics results were provided in Supplementary Table 3.

## Results

### PWI induced more pronounced depression-like behaviors in female adolescent mice than those of males

Both female and male mouse pups were subjected to varying durations of PWI to study sex-specific differences and the influence of isolation duration on depression-like behaviors. Two-way ANOVA revealed that neither the 6-week PWI duration nor sex significantly influenced SPT outcomes (Two-way ANOVA: F(1, 50) = 3.644, *p* = 0.062 for the PWI effect; F(1, 50) = 0.393, *p* = 0.534 for the sex effect) (Supplementary Fig. 1A). However, 6 weeks of PWI significantly affected immobility time in the FST (Two-way ANOVA: F(1, 72) = 17.320, *p* < 0.001 for the PWI effect) (Supplementary Fig. 1B). Post-hoc analyses indicated that 6-week PWI resulted in increased immobility in female mice (*p* < 0.001), but not male mice (*p* = 0.066). Extending the PWI duration to 8 weeks, two-way ANOVA revealed that 8-week PWI duration significantly influenced SPT outcomes, with no significant effect of sex (Two-way ANOVA: F(1, 50) = 19.410, *p* < 0.001 for the PWI effect; F(1, 50) = 0.195, *p* = 0.660 for the sex effect) (Fig. 1A). However, both PWI and sex had significant effects on FST outcomes (Two-way ANOVA: F(1, 72) = 17.340, *p* < 0.001 for the PWI effect; F(1, 72) = 8.500, *p* = 0.005 for the sex effect) (Fig. 1B). Post-hoc results showed that PWI duration to 8 weeks significantly decreased sucrose preference in both female (*p* = 0.028) and male (*p* = 0.001) (Fig. 1A) and increased immobility only in female mice during the FST (*p* < 0.001) (Fig. 1B). These results highlighted sex-specific variations in response to prolonged social isolation, impacting reward sensitivity and depressive-like behavior.

**Fig. 2:**
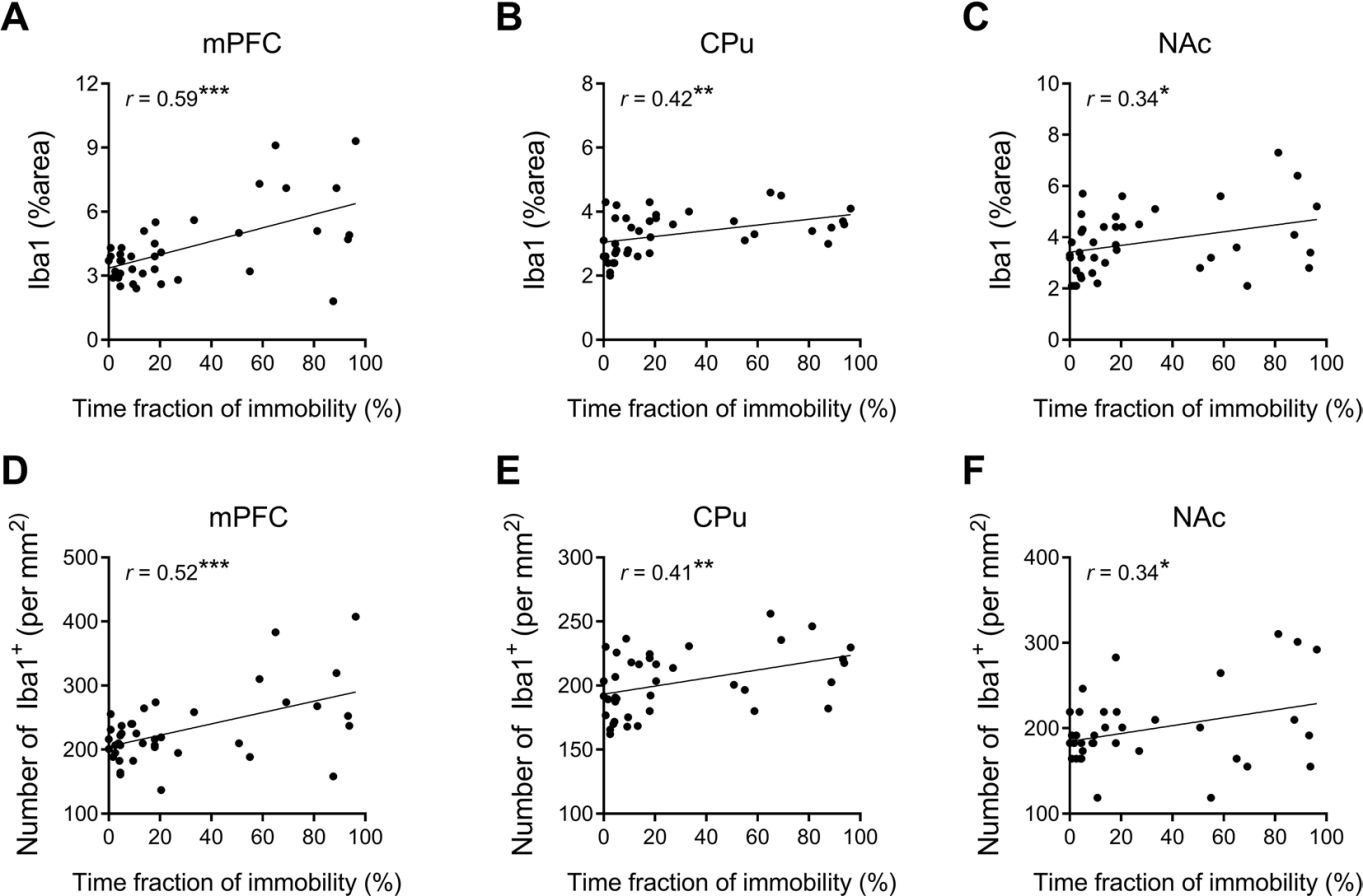
Iba1^+^ area and density are positively correlated with the immobility time in the FST. Correlations between the immobility time in the FST and Iba1^+^ areas (**A**, **B**, and **C**), as well as between the immobility time in the FST and Iba1^+^ cell density (**D**, **E**, and **F**) in the mPFC, CPu, and NAc. The Pearson correlation coefficient (r) and its corresponding significant *p* values (^*^p < 0.05, ^**^p < 0.001, and ^***^p < 0.001) were given for each analysis. Data points represented individual samples, with the dashed line indicating the best-fit line

Given that hormonal fluctuations can influence behaviors [48, 49], we categorized the estrus cycle of these female mice immediately before FST. Mice were grouped into SR (proestrus and estrus) and NR (metestrus and diestrus) and compared their FST scores. The result revealed that the estrus cycle had no discernible influence on FST outcomes (unpaired T-test: *t* = 0.1, degree of freedom (*df*) = 18, *p* = 0.950 for 6w-PWI; *t* = 0.8, *df* = 36, *p* = 0.450 for 8w-PWI) (Supplementary Fig. 2). These findings suggested that adolescent female mice are more susceptible to displaying depression-like behaviors after 8 weeks of PWI compared to males. Consequently, we utilized the 8-week PWI paradigm for subsequent experiments.

### PWI caused microglial activation in the FS areas

We examined the degrees of microglial activation in three FS areas, mPFC, CPu, and NAc after 8 weeks of PWI treatment. Microglia were detected by immunostaining for Iba1, a marker for both resting and activated microglia, and assessed the degree of microglial activation by quantifying the areas (%) with Iba1-immunoreactive signals and the cell densities within the chosen regions [50]. Results showed that 8 weeks of PWI led to a significant increase in the Iba1^+^ area (Two-way ANOVA: F(1, 36) = 22.310, *p* < 0.001 for the PWI effect) (Fig. 1C and D). Post-hoc analysis showed a significant difference between GH and PWI groups in females (*p* < 0.001) but not in males (*p* = 0.447). Similarly, PWI significantly increased cell density (Two-way ANOVA: F(1, 36) = 20.270, *p* < 0.001 for the PWI effect), with a significant increase in females (*p* < 0.001) and no significant difference in males (*p* = 0.822) in the mPFC (Fig. 1C and E). In the CPu, PWI also significantly increased the Iba1^+^ area (Two-way ANOVA: F(1, 36) = 4.598, *p* = 0.001 for the PWI effect) (Fig. 1F and G). However, post-hoc analysis did not show significant differences between GH and PWI groups in either females (*p* = 0.127) or males (*p* = 0.460). Additionally, PWI significantly increased cell density in the CPu (Two-way ANOVA: F(1, 36) = 5.333, *p* = 0.027 for the PWI effect), with a significant increase in females (*p* = 0.046) but not in males (*p* = 0.614) (Fig. 1F and H). Analysis of microglial levels in the NAc showed that PWI did not significantly affect the Iba1^+^ area (Two-way ANOVA: F(1, 36) = 3.130, *p* = 0.085 for the PWI effect), with no significant differences between GH and PWI groups in either females (*p* = 0.401) or males (*p* = 0.379) (Fig. 1I and J). Although PWI had a marginally significant effect on cell density in the NAc (Two-way ANOVA: F(1, 36) = 4.448, *p* = 0.042 for the PWI effect), post-hoc analysis revealed no significant differences between GH and PWI groups in females (*p* = 0.059) or males (*p* = 0.726) (Fig. 1I and K). Collectively, in the CPu (Fig. 1F-H) and NAc (Fig. 1I-K) of female mice, 8 weeks of PWI led to a mild increase in the number of Iba1^+^ cells without significantly affecting the Iba1^+^ area. None of these changes were observed in male mice subjected to 8 weeks of PWI treatment.

To unveil the relationships between microglial activation and depressive-like behaviors, we explored the correlation between the duration of immobility in the FST and the Iba1^+^ area and cell density in three areas related with the FS circuit. The results showed positive correlations between the duration of immobility in the FST and both the Iba1^+^ areas and cell densities in all three FS-related areas. Among these, the mPFC exhibited the highest correlations, with the highest Pearson r values (*r* = 0.59, *p* < 0.001 for microglial cell density; *r* = 0.52, *p* < 0.001 for Iba1^+^ area. Figure 2A, and D). These findings suggested a connection between microglial activation, particularly in the mPFC, and the expression of depressive behaviors.

**Fig. 3:**
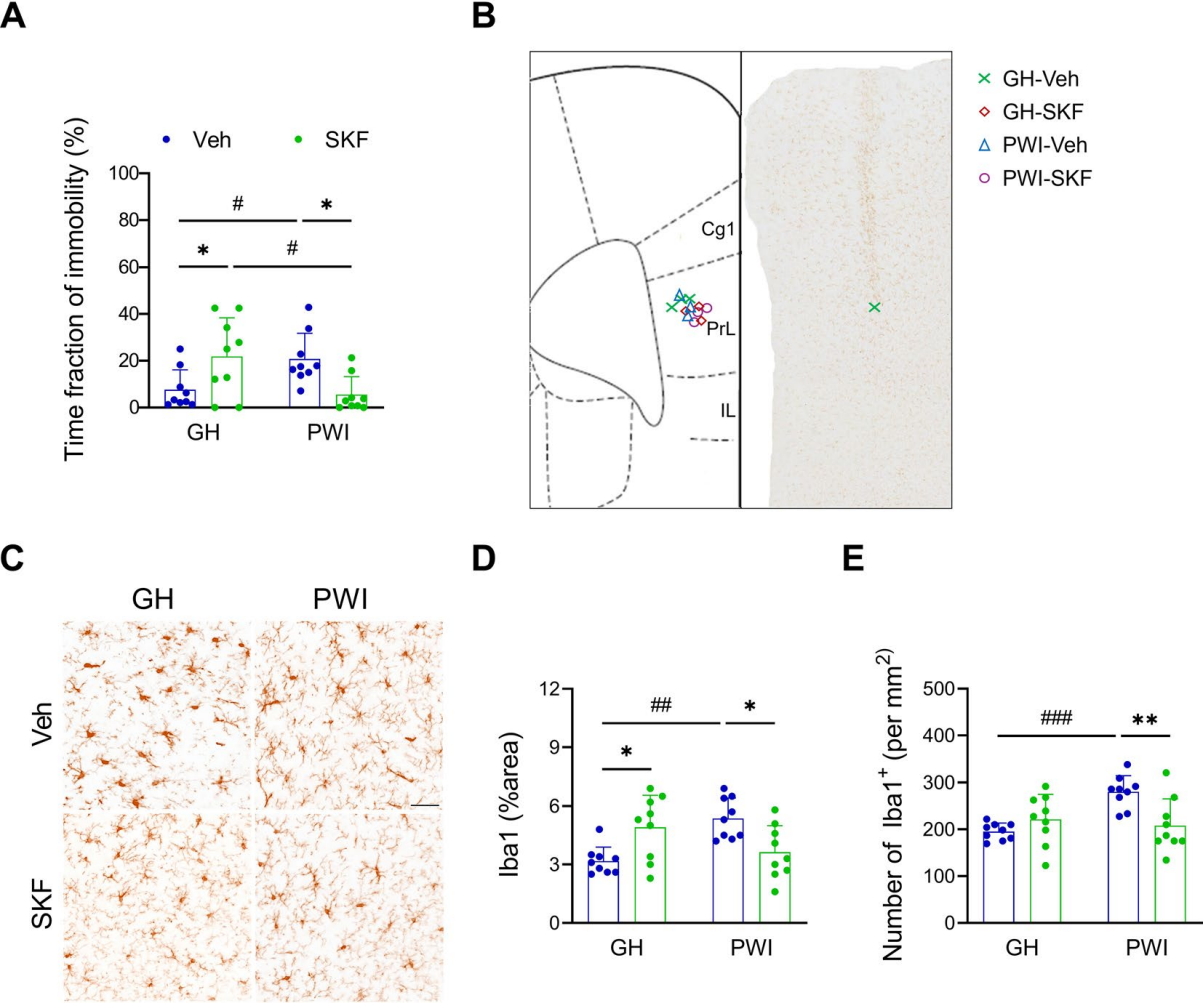
SKF alleviated PWI-induced microglial activation and depressive-like behaviors. (**A**) Quantitative results of the immobility time in FST. (**B**) Representative figure showing the needle trajectory for SKF injection into the prelimbic area of the mPFC. The image on the right depicted the needle tract resulting from stereotaxic surgery aimed at targeting the prelimbic area of the mPFC. The symbols (✕/◇/△/○) on left mark the specific locations of the injection sites. Take three mice as representatives for each group. Cg1: cingulate area 1; PrL: prelimbic cortex; IL: infralimbic cortex. (**C**) Representative micrographs of Iba1 immunostaining in the prelimbic region of the mPFC. (**D**) Quantitative results of Iba1^+^ area in mPFC. (**E**) Quantitative results of Iba1^+^ density in mPFC. *n* = 9 mice. Data were expressed as mean ± SD. Sidak’s multiple comparisons test was used following two-way ANOVA. Post-hoc results showed that ^*^p < 0.05 and ^**^p < 0.01 indicated significant differences between Veh and SKF mice, while ^#^*p* < 0.05, ^##^*p* < 0.01, and ^###^*p* < 0.001 represented significant differences between GH and PWI mice

### Intra-mPFC SKF injection alleviated PWI-induced local microglial activation and depressive-like behaviors

We then determined the relative expression levels of D1R and D2R in the mPFC of PWI mice (Supplementary Fig. 3). Our results demonstrated a significant impact of both 8 weeks of PWI and sex on D2R levels (Two-way ANOVA: F(1, 32) = 5.940, *p* = 0.021 for the PWI effect, F(1, 32) = 4.245, *p* = 0.048 for the PWI effect) (Supplementary Fig. 3A and C). Post-hoc comparisons showed a significant increase in females (*p* = 0.042), while no significant change was observed in males (*p* = 0.529). There were no significant alterations in D1R levels in both PWI effect (Two-way ANOVA: F(1, 32) = 1.197, *p* = 0.282 for the PWI effect) and sex effect (Two-way ANOVA: F(1, 32) = 2.497, *p* = 0.124 for the sex effect) (Supplementary Fig. 3A and B). In comparing the PWI group to the GH group in female mice, there appeared to be a tendency towards a lower D1R/D2R ratio by approximately 20% (Two-way ANOVA: F(1, 32) = 0.985, *p* = 0.328 for the PWI effect, F(1, 32) = 9.407, *p* = 0.004 for the sex effect) (Supplementary Fig. 3A and D). However, these differences were not statistically significant in the post-hoc analysis (*p* = 0.242 for females; *p* = 0.986 for males).

To address the altered D1R/D2R ratio in the mPFC of PWI mice, we considered two potential approaches: inhibiting D2R signaling or enhancing D1R signaling pathways. While D2R antagonists have shown potential in alleviating depression symptoms, their use is associated with significant adverse effects, such as extrapyramidal symptoms and hyperprolactinemia [51–53]. Therefore, we decided to administer the D1-like dopamine receptor agonist SKF into the mPFC of PWI female mice (Fig. 3B). Two-way ANOVA indicated significant interactions between PWI and SKF in the FST immobility time (Two-way ANOVA: F(1, 32) = 14.950, *p* < 0.001) (Fig. 3A), as well as the Iba1^+^ areas (Two-way ANOVA: F(1, 32) = 17.580, *p* < 0.001) (Fig. 3C and D) and cell densities (Two-way ANOVA: F(1, 32) = 11.310, *p* = 0.002) (Fig. 3C and E) in the mPFC. Post-hoc analyses revealed that SKF significantly alleviated the PWI-induced increases in FST immobility time (*p* = 0.016) (Fig. 3A), the Iba1^+^ areas (*p* = 0.011) (Fig. 3C and D), and the Iba1^+^ cell densities (*p* = 0.003) (Fig. 3C and E). However, in naïve GH mice, SKF led to an increase in FST immobility time (*p* = 0.025) (Fig. 3A) and the total Iba1^+^ areas (*p* = 0.011) (Fig. 3C and D).

### SKF modulated inflamagen-elicited inflammatory responses in BV2 microglial cells

**Fig. 4:**
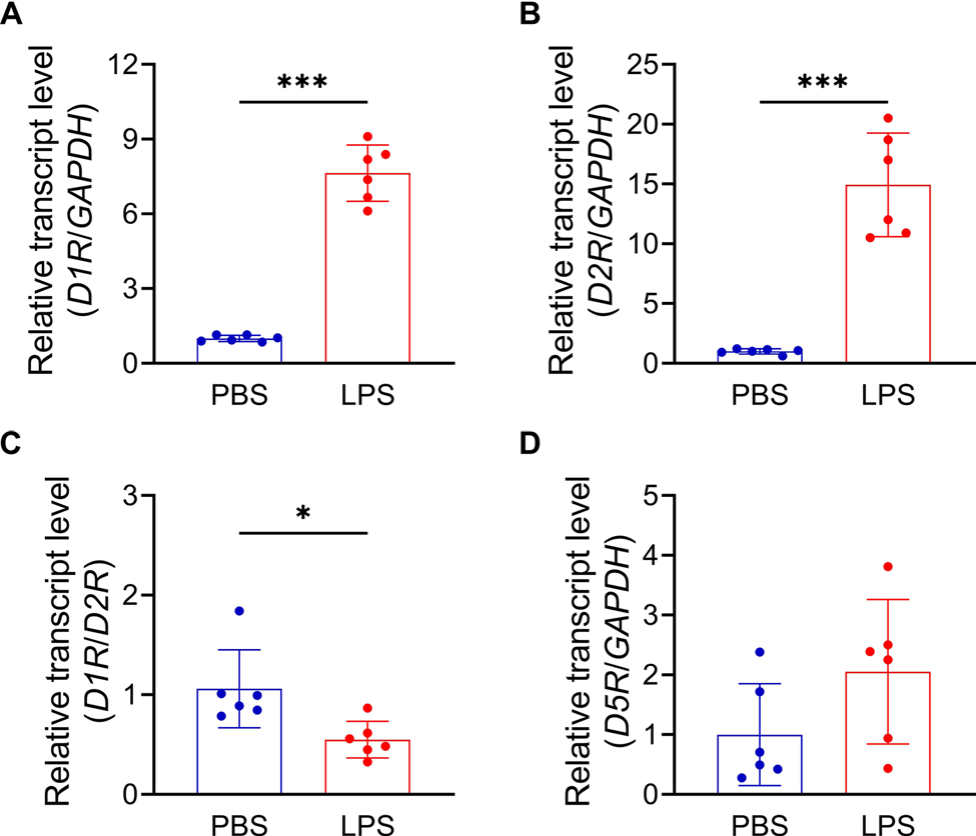
Gene levels of *D1R*, *D2R*, and *D5R* were stimulated LPS treatment in BV2 microglial cells. (**A**) Levels of *D1R*. (**B**) Levels of *D2R*. (**C**) Levels of *D1R/D2R* ratio. (**D**) Levels of *D5R*. *n* = 6. Data were expressed as mean ± SD, and analyzed with an unpaired two-tailed Student’s t-test. ^*^p < 0.05 and ^***^p < 0.001 indicate significant differences compared to PBS treatment

Next, we evaluated the expression of *D1R*, *D2R*, and *D5R* genes in BV2 microglial cells under basal and LPS-stimulated conditions. RT-PCR analysis confirmed the presence of *D1R*, *D2R*, and *D5R* genes in BV2 cells (Fig. 4). Following a 1-hour LPS treatment, mRNA levels of *D1R* (unpaired T-test: *t* = 14.3, *df* = 10, *p* < 0.001) and *D2R* (unpaired T-test: *t* = 7.9, *df* = 10, *p* < 0.001) significantly increased (Fig. 4A and B), whereas *D5R* expression remained unchanged (unpaired T-test: *t* = 1.7, *df* = 10, *p* = 0.111) (Fig. 4D). Furthermore, the D1R/D2R ratio decreased under these inflammatory conditions (unpaired T-test: *t* = 2.9, *df* = 10, *p* = 0.016) (Fig. 4C), consistent with observations in PWI female mice (Supplementary Fig. 3).

**Fig. 5:**
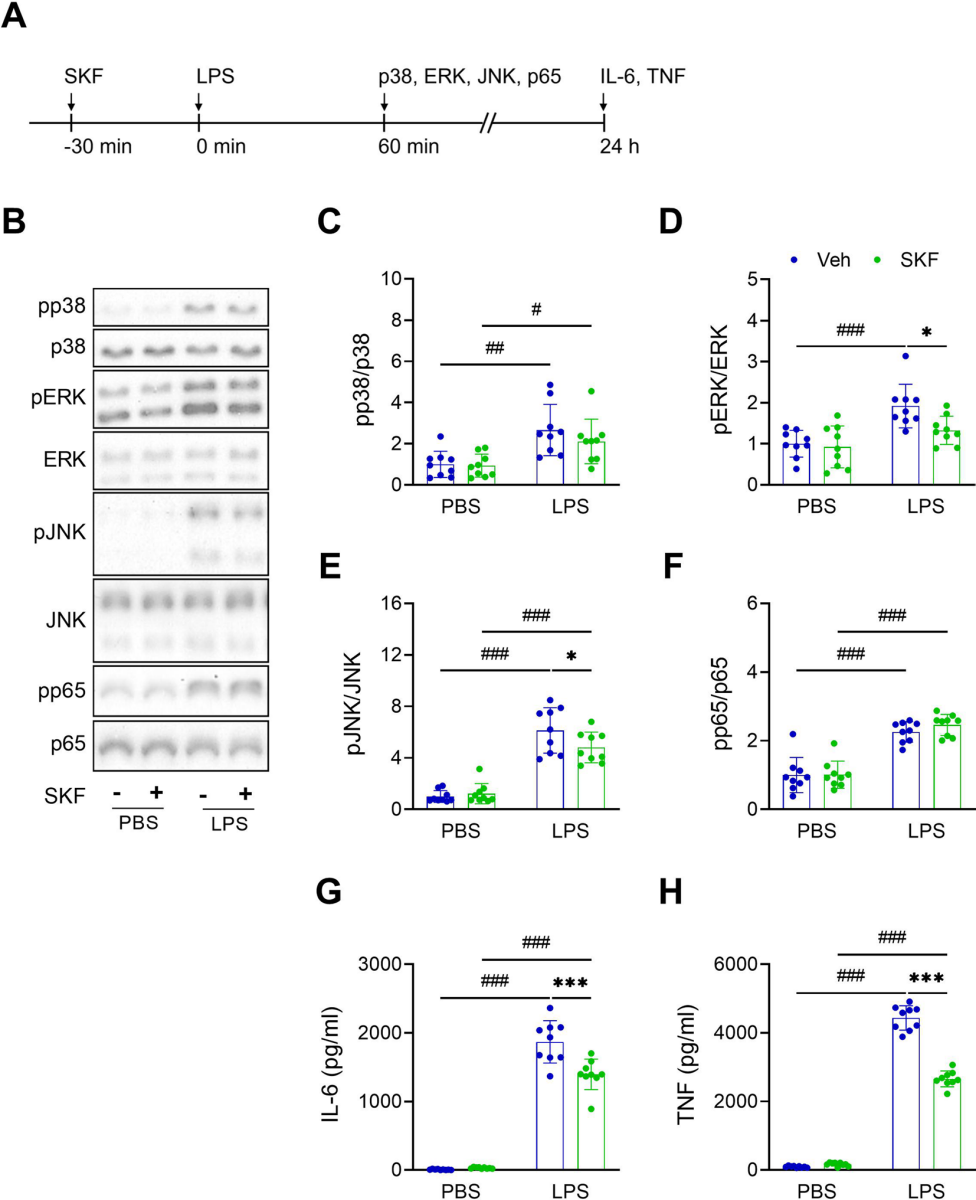
SKF partially blocked LPS-induced inflammatory responses in BV2 microglial cells. (**A**) The experimental timeline. (**B**) Representative Western blot images. (**C**) Levels of pp38. (**D**) Levels of pERK. (**E**) Levels of pJNK. (**F**) Levels of pp65. (**G**) Levels of IL-6 in conditioned media. (**H**) Levels of TNF in conditioned media. *n* = 9. Data were expressed as mean ± SD. Sidak’s multiple comparisons test was used following two-way ANOVA. Post-hoc results showed that ^*^p < 0.05 and ^***^p < 0.001 indicated significant differences between Veh and SKF treatment, while ^#^*p* < 0.05, ^##^*p* < 0.01, and ^###^*p* < 0.001 represented significant differences between PBS and LPS treatment. Please refer to Supplementary Fig. 7 for whole Western blots

Subsequently, we investigated the effect of SKF on microglial inflammatory responses in cultured BV2 microglial cells. D1R, a G protein-coupled receptor, acts as a crucial regulator of the MAPK signaling pathway, involving p38, ERK, and JNK [54]. Considering the crucial role of the MAPK and NF-κB pathways in regulating transcriptional activity to modulate the secretion of pro-inflammatory cytokines [55–58]., we focused our investigation on these signaling cascade. Initially, we found that SKF concentrations exceeding 5 µM induced cell death in BV2 microglial cells (One-way ANOVA: F(3, 32) = 1.613, *p* < 0.001; post-hoc analysis showed *p* = 0.010 compared to 0 µM) (Supplementary Fig. 4). Consequently, we selected the non-toxic dose of 2.5 µM for further investigations (One-way ANOVA: F(3, 32) = 1.613, *p* < 0.001; post-hoc analysis showed *p* = 0.961 compared to 0 µM). To explore the efficacy of LPS-induced inflammatory responses in BV2 microglial cells, we assessed the activation status of NF-κB, the key mediator associated with inflammation [59]. The results showed a dose-dependent trend in NF-κB p65 phosphorylation between 25 and 250 ng/mL following a 1-hour LPS treatment (One-way ANOVA: F(4, 10) = 0.824, *p* = 0.007) (Supplementary Fig. 5). We selected a relatively stable dose of 50 ng/ml to induce the inflammatory response (Post-hoc analysis showed *p* = 0.010 compared to 50 ng/ml). Based on prior observations [59–61], we evaluated the phosphorylation levels of p38 and ERK1/2, JNK and p65 at 1 h following the LPS treatment (Fig. 5A). Furthermore, levels of IL-6 and TNF in the conditioned media were quantified 24 h post-LPS treatment.

Our results showed that LPS induced phosphorylation of p38 (Two-way ANOVA: F(1, 32) = 21.100, *p* < 0.001 for the LPS effect; post-hoc comparisons between PBS and LPS treatment showed *p* = 0.001 in Veh group and *p* = 0.022 in SKF group), ERK (Two-way ANOVA: F(1, 32) = 20.600, *p* < 0.001 for the LPS effect; post-hoc comparisons between PBS and LPS treatment showed *p* < 0.001 in Veh group and *p* = 0.114 in SKF group), JNK (Two-way ANOVA: F(1, 32) = 127.000, *p* < 0.001 for the LPS effect; post-hoc comparisons between PBS and LPS treatment showed *p* < 0.001 in Veh group and *p* < 0.001 in SKF group), and p65 (Two-way ANOVA: F(1, 32) = 109.000, *p* < 0.001 for the LPS effect; post-hoc comparisons between PBS and LPS treatment showed *p* < 0.001 in Veh group and *p* < 0.001 in SKF group) after 1 h (Fig. 5B-F). Administering SKF 30 min prior to the LPS treatment did not affect p38 (Two-way ANOVA: F(1, 32) = 0.983, *p* = 0.329 for SKF effect; post-hoc comparisons between Veh and SKF treatment showed *p* = 0.987 in PBS group and *p* = 0390 in LPS group) (Fig. 5C) and p65 (Two-way ANOVA: F(1, 32) = 0.670, *p* = 0.419 for SKF effect; post-hoc comparisons between Veh and SKF treatment showed *p* = 0.998 in PBS group and *p* = 0479 in LPS group) (Fig. 5F) phosphorylation, but significantly reduced ERK (Two-way ANOVA: F(1, 32) = 5.260, *p* = 0.029 for SKF effect; post-hoc comparisons between Veh and SKF treatment showed *p* = 0.917 in PBS group and *p* = 0.014 in LPS group) (Fig. 5D) and JNK (Two-way ANOVA: F(1, 32) = 2.020, *p* = 0.165 for SKF effect; post-hoc comparisons between Veh and SKF treatment showed *p* = 0.901 in PBS group and *p* = 0.042 in LPS group) (Fig. 5E) phosphorylation levels. Furthermore, LPS exposure significantly increased the production of IL-6 (Two-way ANOVA: F(1, 32) = 649.000, *p* < 0.001 for LPS effect; post-hoc comparisons between PBS and LPS treatment showed *p* < 0.001 in Veh group and *p* < 0.001 in SKF group) (Fig. 5G) and TNF (Two-way ANOVA: F(1, 32) = 2298.000, *p* < 0.001 for LPS effect; post-hoc comparisons between PBS and LPS treatment showed *p* < 0.001 in Veh group and *p* < 0.001 in SKF group) (Fig. 5H) in BV2 microglial cells. Pre-treatment with SKF effectively inhibited the LPS-induced elevation of IL-6 (Two-way ANOVA: F(1, 32) = 12.600, *p* = 0.001 for SKF effect; post-hoc comparisons between Veh and SKF treatment showed *p* = 0.956 in PBS group and *p* < 0.001 in LPS group) and TNF (Two-way ANOVA: F(1, 32) = 143.000, *p* < 0.001 for SKF effect; post-hoc comparisons between Veh and SKF treatment showed *p* = 0.732 in PBS group and *p* < 0.001 in LPS group) levels (Fig. 5G and H).

The anti-inflammation effect of SKF was further validated in TNF-treated BV2 microglial cells using a treatment protocol slightly different from that of LPS (Supplementary Fig. 6A). TNF triggered elevated phosphorylation of p38, ERK, and JNK within 15 min, followed by p65 phosphorylation after 2 h (Supplementary Fig. 6B-F). Pre-treatment with SKF did not inhibit the TNF-induced phosphorylation of p38 (Two-way ANOVA: F(1, 32) = 0.000, *p* = 0.991 for the SKF effect; post-hoc comparisons between Veh and SKF treatment showed *p* = 0.974 in PBS group and *p* = 0.978 in TNF group), ERK (Two-way ANOVA: F(1, 32) = 0.638, *p* = 0.430 for the SKF effect; post-hoc comparisons between Veh and SKF treatment showed *p* = 0.959 in PBS group and *p* = 0.319 in TNF group), and p65 (Two-way ANOVA: F(1, 32) = 0.403, *p* = 0.530 for the SKF effect; post-hoc comparisons between Veh and SKF treatment showed *p* = 0.535 in PBS group and *p* = 0.999 in TNF group) (Supplementary Fig. 6C, D, and F). However, it reduced levels of phosphorylated JNK (Two-way ANOVA: F(1, 32) = 3.267, *p* = 0.080 for the SKF effect; post-hoc comparisons between Veh and SKF treatment showed *p* = 0.980 in PBS group and *p* = 0.047 in TNF group) (Supplementary Fig. 6E). TNF also increased IL-6 production in BV2 cells (Two-way ANOVA: F(1, 32) = 6.210, *p* = 0.018 for the TNF effect; post-hoc comparisons between PBS and TNF treatment showed *p* < 0.001 in Veh group and *p* = 0.052 in SKF group) (Supplementary Fig. 6G). Pre-treatment with SKF mitigated the TNF-induced elevation of IL-6 levels (Two-way ANOVA: F(1, 32) = 2.010, *p* = 0.166 for the SKF effect; post-hoc comparisons between Veh and SKF treatment showed *p* = 0.008 in PBS group and *p* < 0.001 in TNF group) (Supplementary Fig. 6G). Intriguingly, SKF alone increased IL-6 production in PBS-treated BV2 cells (Supplementary Fig. 6G).

**Fig. 6:**
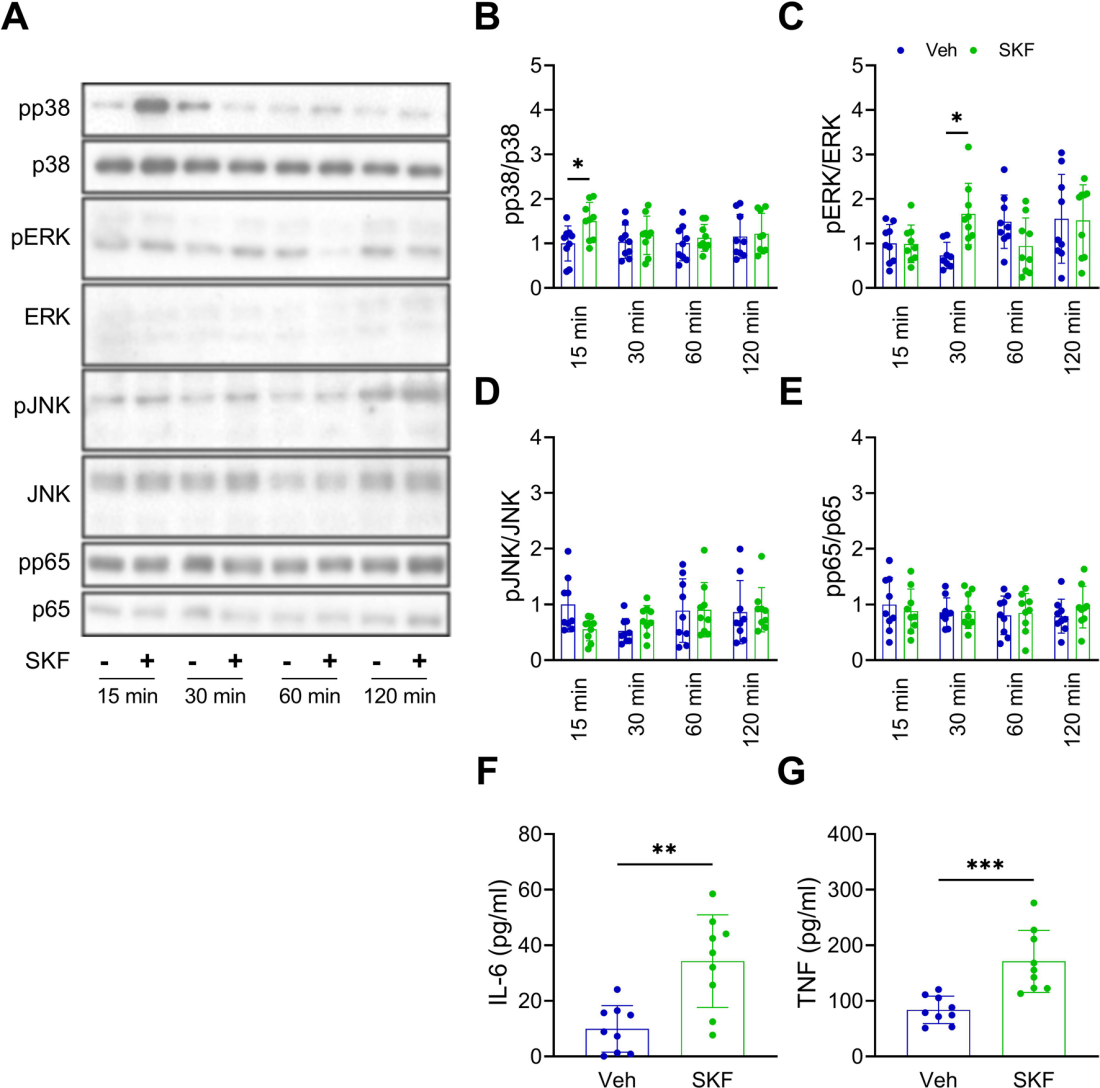
SKF induced inflammatory responses in BV2 microglial cells. (**A**) Representative Western blot images. (**B**) Levels of pp38. (**C**) Levels of pERK. (**D**) Levels of pJNNK. (**E**) Levels of pp65. *n* = 9. Data were expressed as mean ± SD. Sidak’s multiple comparisons test was used following two-way ANOVA. Post-hoc results showed that ^*^p < 0.05 indicated significant differences between Veh and SKF treatment. (**F**) Levels of IL-6 in conditioned media. (**G**) Levels of TNF in conditioned media. *n* = 9. Data were expressed as mean ± SD, analyzed with an unpaired two-tailed Student’s t-test. ^*^p < 0.05, ^**^p < 0.01, and ^***^p < 0.001 indicate significant differences compared to Veh treatment. Please refer to Supplementary Fig. 8 for whole Western blots

These observations led us to re-examine the effect of SKF on cytokine production in naïve BV2 cells. The results showed that SKF induced p38 and ERK phosphorylation at 15 min (Two-way ANOVA: F(3, 64) = 4.533, *p* = 0.037 for the SKF effect; post-hoc comparisons between Veh and SKF treatment showed *p* = 0.048 at 15 min) and 30 min (Two-way ANOVA: F(3, 64) = 0.309, *p* = 0.580 for the SKF effect; post-hoc comparisons between Veh and SKF treatment showed *p* = 0.017 at 30 min), respectively (Fig. 6A-C), while not affecting JNK (Two-way ANOVA: F(3, 64) = 0.238, *p* = 0.627 for the SKF effect) or p65 (Two-way ANOVA: F(3, 64) = 0.118, *p* = 0.733 for the SKF effect) (Fig. 6A, D, and E). Moreover, SKF triggered the secretion of the pro-inflammatory cytokines IL-6 (unpaired T-test: *t* = 3.9, *df* = 16, *p* = 0.001) and TNF (unpaired T-test: *t* = 4.3, *df* = 16, *p* < 0.001) in naïve BV2 cells (Fig. 6F and G).

## Discussion

In this study, we demonstrated that female mice, when exposed to social isolation during their adolescent phase, displayed depression-like behaviors. This phenotype was less pronounced in male mice. In addition to these behavioral changes, social isolation triggered microglial activation in FS areas of female mice, which was also less evident in their male counterparts. Notably, the degree of microglial activation in the mPFC was the most prominent among the three investigated FS areas, and positively correlated with the severity of depression-like behavior. Administering SKF into the mPFC reduced microglial activation and improved depressive behaviors, indicating a potential therapeutic target. This study highlights a female-specific vulnerability to adolescent social isolation, particularly in terms of neuroinflammation and the development of depression.

It is widely recognized that women exhibit higher diagnostic rates, along with distinct symptoms and triggers compared to men [62]. Epidemiological studies have shown significant shifts in depressive symptoms among girls during early adolescence, with prevalence rates of depressive disorders more than twice as high in girls compared to boys by mid-teenage years [63]. Our study confirms that adolescent female mice are more susceptible to social isolation stress and more prone to developing depression-like behaviors. Disturbances in hormonal systems are known to play a role in the development of depression in both humans and mice [64–66]. However, PWI-induced depressive behavior seems independent of the estrus cycle. Other studies have indicated alterations in oxytocin and corticosterone levels in female mice during social isolation [67–69], implying the involvement of hormonal pathways beyond gonadal hormones. The precise mechanism underlying the observed sex effect warrants further investigation in future research.

The SPT and the FST are frequently used behavioral tests in preclinical research to investigate depression-like behaviors and evaluate the effects of potential antidepressant treatments in rodents [70–72]. However, these two behavioral tests probe different emotion-related behaviors and are associated with distinct neural circuits [70, 73]. Alterations in the SPT provide insights into changes in reward and pleasure circuits [73], which are regulated by various brain regions, including the ventral tegmental area, NAc, and PFC [74, 75]. Particularly, the mPFC is associated with decision-making emotional regulation, and reward-related behaviors [76]. On the other hand, the FST, which examines the impact of antidepressant medications and evaluates interventions intended to induce or alleviate depressive-like states [77], primarily delves into the neural circuits associated with coping with stress-inducing situations and the regulation of immobility [71, 72]. The hypothalamic-pituitary-adrenal axis and the serotonergic system are among the neural systems implicated in the FST [78]. Moreover, the PFC, especially the infralimbic and prelimbic regions, is instrumental in controlling stress responses and coping strategies [76, 79], determining whether an animal exhibits active or passive responses during the test [80]. Our study observed that an 8-week PWI induced anhedonia in both male and female mice, as evidenced by changes in sucrose preference in the SPT. However, only female mice displayed increased immobility in the FST. These findings highlight the importance of considering the hormonal and neural circuits and behavioral aspects assessed by the SPT and the FST when studying the impact of psychosocial stress on depression-like behaviors in mice of different sexes.

Studies have consistently found that individuals with depression often exhibit elevated levels of inflammatory markers in their blood and cerebrospinal fluid, including cytokines such as IL-1β, IL-6, and TNF [29–31]. These elevated markers have been associated with depressive symptoms. Chronic stress can activate microglia and trigger inflammatory responses [81]. Increasing evidence indicates that neuroinflammation, including microglial activation, within the FS-related areas plays a pivotal role in the development of depression [82, 83]. Consistent with these findings, we found significant correlations between microglial activation in the FS areas and the severity of depression-like behaviors, especially in the mPFC, highlighting its crucial role in this connection. Neuroinflammation in the FS areas can disrupt neurotransmitter balance, including serotonin, DA, and glutamate, which is associated with depression [84–87]. Microglial activation in these areas can also disrupt nearby neural circuits and impair DA signaling, possibly contributing to mood disorder symptoms [21, 88]. Considering differing roles of microglia in males and females [89, 90], and the link between neuroinflammation and depression, additional research is needed to untangle the causal relationship, particularly in the context of adolescent and sex-specific factors.

It has been demonstrated that both D1R and D5R are expressed in the mPFC neurons [43, 91, 92]. Stimulation of D1R neurons in the mPFC, either optogenetically or chemogenetically, results in rapid and lasting antidepressant effects [43, 91], while inhibition of these neurons blocks the antidepressant effects of ketamine [43]. Additionally, D5R immunoreactivity is present in both excitatory pyramidal neurons and inhibitory interneurons in the mPFC [92]. Activation of D5R in the mPFC increases BDNF expression and signaling, enhances GAD67 expression, and modulates the Akt/GSK-3β pathways [93], although its antidepressant effects still require further validation. Regarding microglia isolated from the mPFC, qPCR analysis reveals *D1R* expression, but not *D5R* [94]. However, our qPCR studies on BV2 cells detected *D5R* mRNA expression, although it did not respond to LPS treatment. Therefore, we consider the contribution of SKF through D5R in the mPFC to be minimal in this study. Given the established role of neuronal D1R in mood disorders [43, 91], we focused on investigating SKF’s direct effects on microglia, particularly as microglial activation in FS areas has been implicated in mood disorders [21, 88]. We acknowledge that further validation of D1R and D5R expression in the mPFC, as well as a more detailed exploration of their role in SKF’s effects, could provide additional insights.

The balance between D1R and D2R in the FS circuit is recognized as essential for mood regulation [39, 40], with an altered D1R/D2R ratio linked to mood disorders [41, 42]. We found that activation of D1-like dopamine receptors alleviated PWI-induced depression-like behaviors and microglial activation in the mPFC of female mice. D1R activation is known to modulate the activity of different types of neurons in the mPFC, including pyramidal neurons and interneurons [43]. This modulation can affect the balance of excitatory and inhibitory neurotransmission in the mPFC, which is critical for mood regulation [95, 96]. Dopaminergic modulation in the mPFC not only mitigate the HPA axis response of chronic stress on mood [97], also modulate neuroinflammation [94]. D1R agonists have demonstrated anti-inflammatory effects in the central nervous system [18, 98]. Hence, we suggest that the antidepressant effect of SKF is partially attributable to D1-like dopamine receptor-mediated anti-microglial activation.

Administering SKF into the mPFC of naïve mice led to local microglial activation and the development of depression-like behaviors. It is possible that SKF disrupted the D1R/D2R balance in these mice, subsequently triggering microglial activation and the onset of depression. Given the established role of neuronal D1R in mood disorders [43, 99] and the identification of all five subtypes of DA receptors on microglia [27, 100–102], we further investigated the impact of SKF on BV2 microglial cells, as microglial activation in FS areas is implicated in mood disorder [21, 88]. The in vitro findings confirmed that, under conditions of inflamagen stimulation, SKF appears to suppress pro-inflammatory cytokine production probably via the JNK-dependent pathway. On the other hand, in the absence of stimulation, SKF triggered pro-inflammatory responses, including increased p38 and ERK phosphorylation and levels of secreted IL-6 and TNF. These results prompt further investigation into the intricate and context-dependent actions of D1-like dopamine receptors in modulating microglial responses and depression.

It is known that pro-inflammatory cytokines are regulated by signaling cascades, including the MAPK and NF-κB pathways [55–58]. MAPK pathways involve JNK and ERK, which activate transcription factors promoting cytokine production [54]. NF-κB p65 activation involves phosphorylation, enabling its nuclear translocation and initiation of pro-inflammatory gene transcription [103]. In this study, SKF inhibits phosphorylation of JNK and ERK, disrupting the MAPK pathway and reducing activation of downstream transcription factors like AP-1, essential for cytokine gene expression. By targeting pJNK and pERK, the drug reduces cytokine production driven by MAPK signaling. However, it does not inhibit NF-κB p65 phosphorylation, leaving NF-κB-regulated cytokine production unaffected. Furthermore, recent studies have shown that D1R activation has anti-inflammatory effects in the brain and periphery. D1R agonists suppress neuroinflammation by inhibiting microglial production of pro-inflammatory cytokines like IL-1β and TNF [94, 104]. This anti-inflammatory action is mediated through the inhibition of nucleotide-binding oligomerization domain 3 (NLRP3) inflammasome activation, involving increased ubiquitylation and autophagosomal degradation of NLRP3 [104, 105]. These effects are associated with improved neurological outcomes in models of intracerebral hemorrhage and Parkinson’s disease. The anti-inflammatory properties of D1R agonists suggest their potential as therapeutic agents for various inflammatory conditions, including delirium, neurodegenerative diseases [94, 104, 105].

Further exploration is warranted regarding the dual function demonstrated by D1 receptors in regulating inflammatory responses. Given that D1R is a type of G protein-coupled receptor, which acts as a crucial upstream regulator of the MAPKKK/MAPK signaling pathway, we propose that the conflicting roles of D1R in inflammation may arise from interaction among various MAPK signaling pathways. Previous research has demonstrated that D1R regulates multiple voltage-gated ion channels, NMDA receptors, and the MAPK signaling pathway, including ERK, JNK, and p38 [54]. Our investigation primarily focuses on the MAPK signaling pathway, a central route in inflammation. Our results elucidate the dual nature of D1R. In activated microglia or under inflammatory conditions (e.g., exposure to LPS or TNF), the activation of D1-like dopamine receptors inhibits the production of pro-inflammatory cytokines, such as IL-6 and TNF, likely through suppression of JNK signaling phosphorylation, consistent with previous studies [106–108]. Conversely, in untreated microglia, the activation of D1-like dopamine receptors directly stimulates the p38 and ERK pathways, resulting in the production of IL-6 and TNF, aligning with previous observations that SKF induces striatal neuronal progenitor cell death via a p38 and ERK dependent mechanism [54, 109]. Additionally, SKF-38,393, another D1R agonist, has been shown to induce cytotoxicity and oxidative stress in SK-N-MC neuroblastoma cells by activating the ERK signaling pathway [54]. Hence, we propose that the contradictory functions of D1R in inflammation may arise from the activation status of different MAPK signaling pathways.

This study has several limitations. First, the most appropriate behavioral tests for assessing depressive phenotypes in rodent models remain a subject of debate. In this study, we utilized the SPT and the FST as the primary behavioral assessments. However, while the FST was originally designed to evaluate antidepressant efficacy, its validity as a reliable measure of depression induction has been increasingly questioned [110]. Second, although prior studies confirm the expression of D1R and D5R in mPFC neurons, this study did not directly validate the expression of these receptors in vivo within the experimental context. The absence of cell-specific receptor expression profiling in the mPFC may limit the interpretation of SKF’s effects. Third, while the study primarily investigates the effects of SKF on microglia, the potential contributions of other cell types, such as astrocytes and neurons, to the observed outcomes were not explored. This narrower focus may overlook additional mechanisms of SKF effects. These limitations should be taken into consideration when interpreting our findings.

## Conclusions

We have observed that female mice, when socially isolated during adolescence, exhibit depression-like behaviors, whereas this phenotype is less evident in their male counterparts. Among the three investigated FS areas, the mPFC displays the most pronounced microglial activation, which positively correlated with the severity of depression-like behaviors. Administration of a D1-like dopamine receptor agonist in the mPFC reduces microglial activation and ameliorates depression-like symptoms in female PWI mice. However, this treatment also induces microglial activation and depression-like behaviors in non-isolated mice. These findings collectively emphasize a sex-specific susceptibility to the consequences of social isolation during adolescence, particularly in terms of neuroinflammation and the development of depression. Moreover, although stimulating the D1-like dopamine receptors signaling pathway shows promise in mitigating social isolation-induced neuroinflammation and depression, the potential risk of inducing side effects by SKF in a balanced D1R/D2R situation deserves further investigation.

## Electronic supplementary material

Below is the link to the electronic supplementary material.

Supplementary Material 1

## Data Availability

No datasets were generated or analysed during the current study.

## Abbreviations

CPu: caudate/putamen
D1R: dopamine D1 receptor
D2R: dopamine D2 receptor
D5R: dopamine D5 receptor
DA: dopamine
DMEM/F12: Dulbecco’s Modified Eagle Medium F12
FS: frontostriatal
FST: forced swimming test
GH: group-housed
LPS: lipopolysaccharide
mPFC: medial prefrontal cortex
NAc: nucleus accumbens
NR: non-receptive
PBS: phosphate buffered saline
PWI: post-weaning isolation
SKF: SKF-81297
SR: sexually receptive
SPT: sucrose preference test
TNF: tumor necrosis factor
Veh: Vehicle
