# Supplementary material for: Dopamine D1 receptor agonist alleviates post-weaning isolation-induced neuroinflammation and depression-like behaviors in female mice
Source: Behav Brain Funct. 2025 Mar 10;21:6. doi: 10.1186/s12993-025-00269-y (PMC11895232; doi:10.1186/s12993-025-00269-y)
Supplement: Supplementary file 1 — Supplementary Material 1 [file 12993_2025_269_MOESM1_ESM.docx]

**Supplementary Table 1.** **Details of animals used in this study.**

| **Major Treatment** | **Number of Animals Used** | **Experiments, Procedures, and Number of Animals Presented in datasets** | | | | | | |
| --- | --- | --- | --- | --- | --- | --- | --- | --- |
| 6-week PWI | Set 1: n = 10 mice. 4 groups: GH-Female, GH-Male, PWI-Female, PWI-Male. | SPT: 1 day after the end of 6-week PWI. (**Supplementary Fig. 1A**) | | Estrus cycle: before FST conduct. (**Supplementary Fig. 2B)** | | | FST: 2 day after the end of 6-week PWI. (**Supplementary Fig. 1B**) | |
|  | Set 2: n = 9 mice. 4 groups: GH-Female, GH-Male, PWI-Female, PWI-Male. | SPT: 1 day after the end of 6-week PWI. (**Supplementary Fig. 1A**) | | | | FST: GH = 10 mice; PWI = 10 mice, 2 day after the end of 6-week PWI. (**Supplementary Fig. 1B**) | | |
| 8-week PWI | Set 1: n = 10 mice. 4 groups: GH-Female, GH-Male, PWI-Female, PWI-Male. | SPT: 1 day after the end of 8-week PWI. (**Fig. 1A**) | Estrus cycle: before FST conduct. (**Supplementary Fig. 2C**) | | FST: 2 day after the end of 8-week PWI. (**Fig. 1B**) | | | Immunohistochemistry: 3 day after the end of 8-week PWI. (**Fig. 1C-K**) |
|  |  |  |  |  |  |  |  | Corrlation FST and microglial expression: total 38 mice (**Fig. 2A-F**) |
|  | Set 2: n = 9 mice. 4 groups: GH-Female, GH-Male, PWI-Female, PWI-Male. | SPT: 1 day after the end of 8-week PWI. (**Fig. 1A**) | Estrus cycle: before FST conduct. (**Supplementary Fig. 2C**) | | FST: 2 day after the end of 8-week PWI. (**Fig. 1B**) | | | western: GH = 9 mice; PWI = 9 mice, 3 day after the end of 8-week PWI. (**Supplementary Fig. 3**) |
| 8-week PWI + intra-PFC infusion of SKF treatment | Set 1: n = 9 mice. 4 groups: GH-Veh, GH-SKF, PWI-Veh, PWI-SKF. | FST: 2 day after the end of 8-week PWI. (**Fig. 3A**) | | | | IHC: 3 day after the end of 8-week PWI. (**Fig. 3C-E**) | | |

**Supplementary Table 2.** **Details of antibody information.**

| **primary antibody** | **manufacturer** | **catalog number** | **dilution factor** | **scondary antibody** | **manufacturer** | **catalog number** | **dilution factor** |
| --- | --- | --- | --- | --- | --- | --- | --- |
| D1R | Atlas Antibodies, Stockholm, Sweden | HPA017304 | 1:1000 | goat anti-rabbit IgG | Jackson ImmunoResearch, West Grove, PA, USA | 111-035-003 | 1:10000 |
| D2R | Merck-Millipore, Billerica, MA, USA | AB5084P |  |  |  |  | 1:5000 |
| β-actin |  | AB1501R | 1:10000 | goat anti-mouse IgG |  | 115-035-003 | 1:20000 |
| pp38 | Cell Signaling Technology, Beverly, MA, USA | 9211 | 1:1000 | goat anti-rabbit IgG  goat anti-rabbit IgG |  | 111-035-003 | 1:2000 |
| p38 |  | 8690 |  |  |  |  | 1:20000 |
| pERK |  | 9101 |  |  |  |  |  |
| ERK |  | 9102 |  |  |  |  |  |
| pJNK |  | 4668 |  |  |  |  | 1:2000 |
| JNK |  | 9252 |  |  |  |  | 1:5000 |
| pp65 |  | 3033 |  |  |  |  |  |
| p65 |  | 8242 |  |  |  |  |  |
| Iba1 | Wako Pure Chemical Industries, Osaka, Japan | 019-19741 | 1:1000 |  |  |  | 1:1000 |

**Supplementary Table 3. Details of statistics results.**

| **statistical method: two-way ANOVA followed by Sidak's multiple comparisons test** | | | |
| --- | --- | --- | --- |
| **Panel** | **F(DF_n_, DF_t_) = Interaction, Effect1, Effect2** | ***p* value of two-way ANOVA** | ***p* value of post-hoc** |
| Fig. 1A | F(1, 50) = 0.655, 0.195 (sex), 19.410 (PWI). | Interation: *p* = 0.422; sex effect: *p* = 0.660; PWI effect: *p* < 0.001. | GH-PWI: Female (*p* = 0.028), male (*p* = 0.001); Female-Male: GH (*p* = 0.970), PWI (*p* = 0.446). |
| Fig. 1B | F(1, 72) = 4.202, 8.500 (sex), 17.340 (PWI). | Interation: *p* = 0.044; sex effect: *p* = 0.005; PWI effect: *p* < 0.001. | GH-PWI: Female (*p* < 0.001), male (*p* = 0.250); Female-Male: GH (*p* = 0.791), PWI (*p* = 0.002). |
| Fig. 1D | F(1, 36) = 9.552, 14.540 (sex), 22.310 (PWI). | Interation: *p* = 0.004; sex effect: *p* < 0.001; PWI effect: *p* <0.001. | GH-PWI: Female (*p* < 0.001), male (*p* = 0.447); Female-Male: GH (*p* = 0.850), PWI (*p* < 0.001). |
| Fig. 1E | F(1, 36) = 13.760, 2.550 (sex), 20.270 (PWI). | Interation: *p* < 0.001; sex effect: *p* = 0.119; PWI effect: *p* < 0.001. | GH-PWI: Female (*p* < 0.001), male (*p* = 0.822); Female-Male: GH (*p* = 0.267), PWI (*p* = 0.001). |
| Fig. 1G | F(1, 36) = 0.295, 17.660 (sex), 4.598 (PWI). | Interation: *p* = 0.590; sex effect: *p* < 0.001; PWI effect: *p* = 0.039. | GH-PWI: Female (*p* = 0.127), male (*p* = 0.460); Female-Male: GH (*p* = 0.028), PWI (*p* = 0.004). |
| Fig. 1H | F(1, 36) = 1.100, 10.550 (sex), 5.333 (PWI). | nteration: *p* = 0.301; sex effect: *p* = 0.003; PWI effect: *p =* 0.027. | GH-PWI: Female (*p* = 0.046), male (*p* = 0.614); Female-Male: GH (*p* = 0.241), PWI (*p* = 0.009). |
| Fig. 1J | F(1, 36) = 0.001, 8.424 (sex), 3.130 (PWI). | Interation: *p* = 0.978; sex effect: *p* = 0.006; PWI effect: *p* = 0.085. | GH-PWI: Female (*p* = 0.401), male (*p* = 0.379); Female-Male: GH (*p* = 0.089), PWI (*p* = 0.097). |
| Fig. 1K | F(1, 36) = 1.191, 5.768 (sex), 4.448 (PWI). | Interation: *p* = 0.282;sex effect: *p* = 0.022; PWI effect: *p* = 0.042. | GH-PWI: Female (*p* = 0.059), male (*p* = 0.726); Female-Male: GH (*p* = 0.591), PWI (*p* = 0.036). |
| Fig. 3A | F(1, 32) = 14.950, 0.181 (PWI), 0.019 (SKF). | Interation: *p* < 0.001; PWI effect: *p* = 0.674; SKF effect: *p* = 0.892. | Veh-SKF: GH (*p* = 0.025), PWI (*p* = 0.016); GH-PWI: Veh (*p* = 0.041), SKF (*p* = 0.010). |
| Fig. 3D | F(1, 32) = 17.580, 1.214 (PWI), 0.000 (SKF). | Interation: *p* < 0.001; PWI effect: *p* = 0.279; SKF effect: *p* > 0.999. | Veh-SKF: GH (*p* = 0.011), PWI (*p* = 0.011); GH-PWI: Veh (*p* = 0.001), SKF (*p* = 0.071). |
| Fig. 3E | F(1, 32) = 11.310, 6.112 (PWI), 2.500 (SKF). | Interation: *p* = 0.002; PWI effect: *p* = 0.019; SKF effect: *p* = 0.124. | Veh-SKF: GH (*p* = 0.387), PWI (*p* = 0.003); GH-PWI: Veh (*p* < 0.001), SKF (*p* = 0.782). |
| Fig. 5C | F(1, 32) = 0.613, 21.100 (LPS), 0.983 (SKF) | Interation: *p* = 0.439; LPS effect: *p* < 0.001; SKF effect: *p* = 0.329. | Veh-SKF: PBS (*p* = 0.987), LPS (*p* = 0.390); PBS-LPS: Veh (*p* = 0.001), SKF (*p* = 0.022). |
| Fig. 5D | F(1, 32) = 3.120, 20.600 (LPS), 5.260 (SKF) | Interation: *p* = 0.087; LPS effect: *p* < 0.001; SKF effect: *p* = 0.029. | Veh-SKF: PBS (*p* = 0.917), LPS (*p* = 0.014); PBS-LPS: Veh (*p* < 0.001), SKF (*p* = 0.114). |
| Fig. 5E | F(1, 32) = 4.000, 127.000 (LPS), 2.020 (SKF) | Interation: *p* = 0.054; LPS effect: *p* < 0.001; SKF effect: *p* = 0.165. | Veh-SKF: PBS (*p* = 0.901), LPS (*p* = 0.042); PBS-LPS: Veh (*p* < 0.001), SKF (*p* < 0.001). |
| Fig. 5F | F(1, 32) = 0.550, 109.000 (LPS), 0.670 (SKF) | Interation: *p* = 0.464; LPS effect: *p* < 0.001; SKF effect: *p* = 0.419. | Veh-SKF: PBS (*p* = 0.998), LPS (*p* = 0.479); PBS-LPS: Veh (*p* < 0.001), SKF (*p* < 0.001). |
| Fig. 5G | F (1, 32) = 15.500, 649.000 (LPS), 12.600 (SKF) | Interation: *p* < 0.001; LPS effect: *p* < 0.001; SKF effect: *p* = 0.001. | Veh-SKF: PBS (*p* = 0.956), LPS (*p* < 0.001); PBS-LPS: Veh (*p* < 0.001), SKF (*p* < 0.001). |
| Fig. 5H | F(1, 32) = 169.000, 2298.000 (LPS), 143.000 (SKF) | Interation: *p* < 0.001; LPS effect: *p* < 0.001; SKF effect: *p* < 0.001. | Veh-SKF: PBS (*p* = 0.732), LPS (*p* < 0.001); PBS-LPS: Veh (*p* < 0.001), SKF (*p* < 0.001). |
| Fig. 6B | F(3, 64) = 1.053, 0.713 (time), 4.533 (SKF). | Interation: *p* = 0.375; time effect: *p* = 0.548; SKF effect: *p* 0.037. | Veh-SKF: 15 min (*p* = 0.048), 30 min (*p* = 0.917), 60 min ( *p* = 0.955), 120 min ( *p* = 0.996) |
| Fig. 6C | F(3, 64) = 3.928, 2.153 (time), 0.309 (SKF). | Interation: *p* = 0.012; time effect: *p* = 0.102; SKF effect: *p* 0.580. | Veh-SKF: 15 min (*p* > 0.999), 30 min (*p* = 0.017), 60 min ( *p* = 0.278), 120 min ( *p* > 0.999) |
| Fig. 6D | F(3, 64) = 1.876, 1.613 (time), 0.238 (SKF). | Interation: *p* = 0.143; time effect: *p* = 0.195; SKF effect: *p* 0.627. | - |
| Fig. 6E | F(3, 64) = 0.442, 0.298 (time), 0.118 (SKF). | Interation: *p* = 0.724; time effect: *p* = 0.827; SKF effect: *p* 0.733. | - |
| Supplementary Fig. 1A | F(1, 50) = 2.572, 0.393 (sex), 3.644 (PWI). | Interation: *p* = 0.115; sex effect: *p* = 0.534; PWI effect: *p* = 0.062. | - |
| Supplementary Fig. 1B | F(1, 72) = 1.204, 7.863 (sex), 17.320 (PWI). | Interation: *p* = 0.276; sex effect: *p* = 0.007; PWI effect: *p* < 0.001. | GH-PWI: Female (*p* < 0.001), male (*p* = 0.066); Female-Male: GH (*p* = 0.409), PWI (*p* = 0.015). |
| Supplementary Fig. 3B | F(1, 32) = 0.004, 2.497 (sex), 1.197 (PWI). | Interation: *p* = 0.948; sex effect: *p* = 0.124; PWI effect: *p* = 0.282. | - |
| Supplementary Fig. 3C | F(1, 32) = 0.978, 4.245 (sex), 5.940 (PWI). | Interation: *p* = 0.330; sex effect: *p* = 0.048; PWI effect: *p* = 0.021. | GH-PWI: Female (*p* = 0.042), male (*p* = 0.529); Female-Male: GH (*p* = 0.076), PWI (*p* = 0.702). |
| Supplementary Fig. 3D | F(1, 32) = 1.460, 9.407 (sex), 0.985 (PWI). | Interation: *p* = 0.236; sex effect: *p* = 0.004; PWI effect: *p* = 0.328. | GH-PWI: Female (*p* = 0.242), male (*p* = 0.986); Female-Male: GH (*p* = 0.010), PWI (*p* = 0.357). |
| Supplementary Fig. 6C | F(1, 32) = 0.077, 144.000 (TNF), 0.000 (SKF) | Interation: *p* = 0.783; TNF effect: *p* < 0.001; SKF effect: *p* = 0.991. | Veh-SKF: PBS (*p* = 0.974), TNF (*p* = 0.978); PBS-TNF: Veh (*p* < 0.001), SKF (*p* < 0.001). |
| Supplementary Fig. 6D | F(1, 32) = 1.350, 10.100 (TNF), 0.638 (SKF) | Interation: *p* = 0.253; TNF effect: *p* = 0.003; SKF effect: *p* = 0.430. | Veh-SKF: PBS (*p* = 0.959), TNF (*p* = 0.319); PBS-TNF: Veh (*p* = 0.009), SKF (*p* = 0.302). |
| Supplementary Fig. 6E | F(1, 32) = 2.405, 210.200 (TNF), 3.267 (SKF) | Interation: *p* = 0.131; TNF effect: *p* < 0.001; SKF effect: *p* = 0.080. | Veh-SKF: PBS (*p* = 0.980), TNF (*p* = 0.047); PBS-TNF: Veh (*p* < 0.001), SKF (*p* < 0.001). |
| Supplementary Fig. 6F | F(1, 32) = 0.339, 24.600 (TNF), 0.403 (SKF) | Interation: *p* = 0.565; TNF effect: *p* < 0.001; SKF effect: *p* = 0.530. | Veh-SKF: PBS (*p* = 0.535), TNF (*p* = 0.999); PBS-TNF: Veh (*p* < 0.001), SKF (*p* = 0.008). |
| Supplementary Fig. 6G | F(1, 32) = 33.500, 6.210 (TNF), 2.010(SKF) | Interation: *p* < 0.001; TNF effect: *p* = 0.018; SKF effect: *p* = 0.166. | Veh-SKF: PBS (*p* = 0.008), TNF (*p* < 0.001); PBS-TNF: Veh (*p* < 0.001), SKF (*p* = 0.052). |
| **statistical method: one-way ANOVA followed by Dunnett's multiple comparisons test** | | | |
| **Panel** | **F(DF_n_, DF_t_)** | ***p* value of one-way ANOVA** | ***p* value of post-hoc** |
| Supplementary Fig. 4 | F(3, 32) = 1.613 | *p* < 0.001 | 0-2.5: p = 0.961, 0-5.0: p = 0.010, 0-10.0: p < 0.001 |
| Supplementary Fig. 5 | F(4, 10) = 0.824 | *p* = 0.007 | 0-25: p = 0.040, 0-50: p = 0.010, 0-100: p = 0.004, 0-250: p = 0.006 |
| **statistical method: unpaired T-test** | | | |
| **Panel** | ***t* value, degree of freedom (*df*)** | ***p* value** | |
| Fig. 4A | *t* = 14.3, *df* = 10 | *p* < 0.001 | |
| Fig.4B | *t* = 7.9, *df* = 10 | *p* < 0.001 | |
| Fig.4C | *t* = 2.9, *df* = 10 | *p* = 0.016 | |
| Fig. 4D | *t* = 1.7, *df* = 10 | *p* = 0.111 | |
| Fig. 6F | *t* = 3.9, *df* = 16 | *p* = 0.001 | |
| Fig. 6G | *t* = 4.3, *df* = 16 | *p* < 0.001 | |
| Supplementary Fig. 2B | *t* = 0.1, *df* = 18 | *p* = 0.950 | |
| Supplementary Fig. 2C | *t* = 0.8, *df* = 36 | *p* = 0.450 | |
| **statistical method: Pearson correlarion** | | | |
| **Panel** | ***r* value** | ***p* value** | |
| Fig. 2A | *r* = 0.59 | *p* < 0.001 | |
| Fig. 2B | r = 0.42 | *p* = 0.006 | |
| Fig. 2C | *r* = 0.34 | *p* = 0.033 | |
| Fig. 2D | *r* = 0.52 | *p* < 0.001 | |
| Fig. 2E | *r* = 0.41 | *p* = 0.008 | |
| Fig. 2F | *r* = 0.34 | *p* = 0.033 | |

**
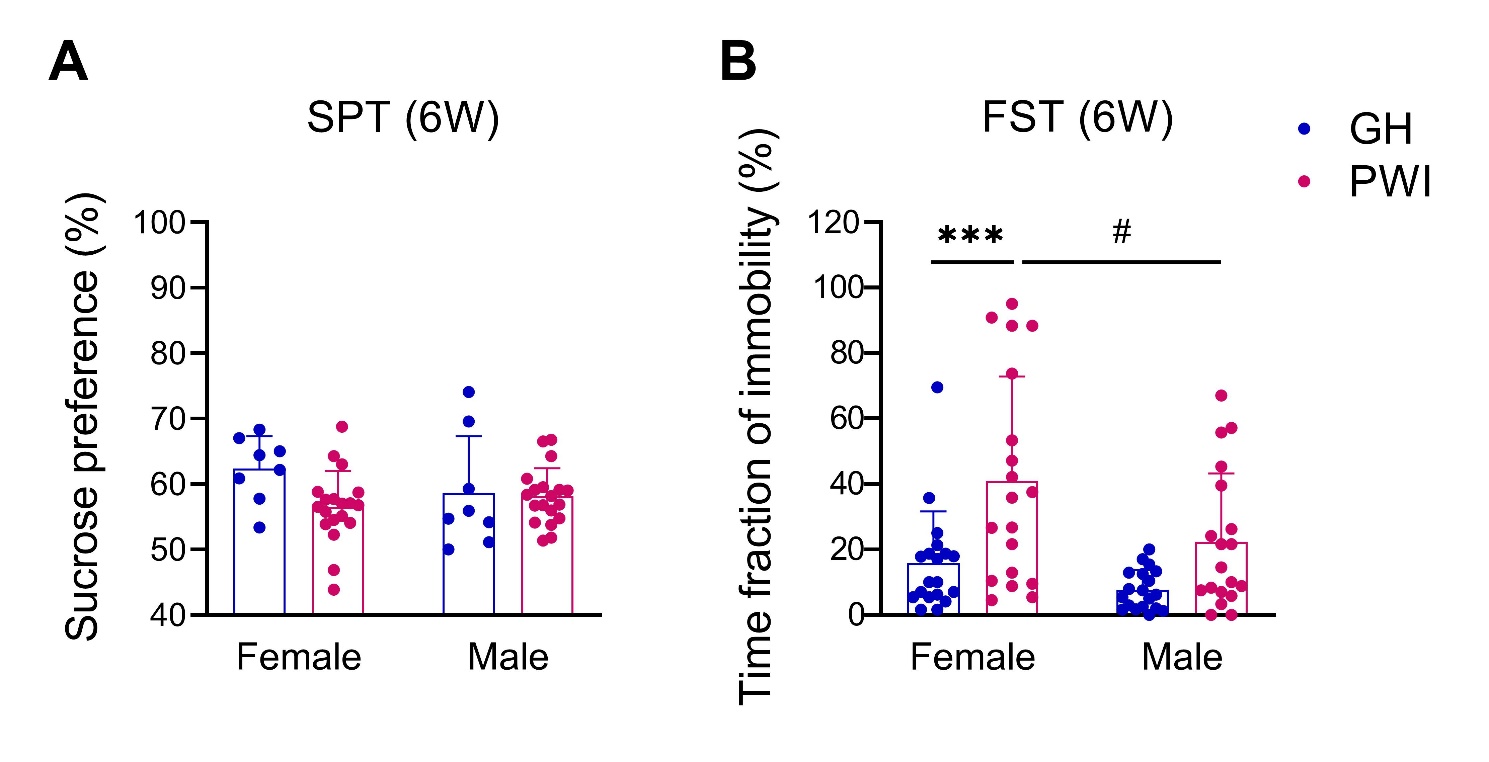
**

**Supplementary Figure 1. Six-week PWI induced induced depression-like behaviors in mice**. (**A**) Results of sucrose preference in SPT after 6 weeks of PWI. n = 8 cages of GH and 19 cages of PWI. (**B**) Results of the exhibition of immobility in FST after 6 weeks of PWI. n = 19 mice. Data were expressed as mean ± SD. Sidak’s multiple comparisons test was used following two-way ANOVA. Post-hoc results showed that  ^***^*p* < 0.001 indicated significant differences between GH and PWI mice, while ^#^*p* < 0.05 represented significant differences between female and male mice.

**
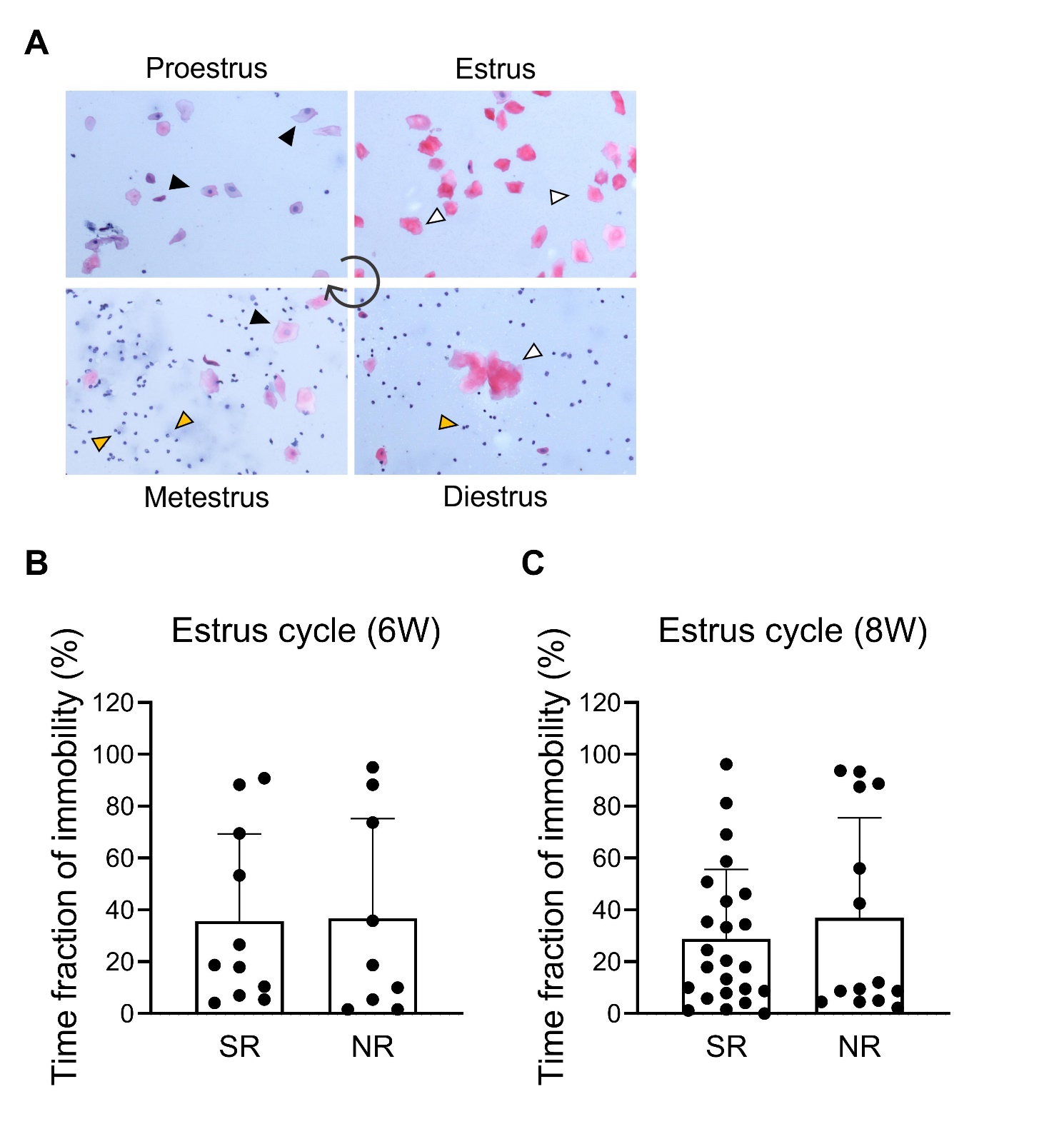
**

**Supplementary Figure 2. Effects of the estrus cycle on the immobility time of the FST**. (**A**) Representative micrograph of vaginal smears stained with Hematoxylin and Eosin. Yellow arrows point to leukocytes, black arrows point to cornified epithelial cells, white arrows point to nucleated epithelial cells. (**B** and **C**) Results of FST results following 6-week and 8-week GH and PWI in 20 and 40 female mice, respectively, grouped into sexually receptive (SR) (proestrus and estrus) and non-receptive (NR) (metestrus and diestrus) stages. Data were expressed as mean ± SD. Statistical analysis performed using unpaired T-test.


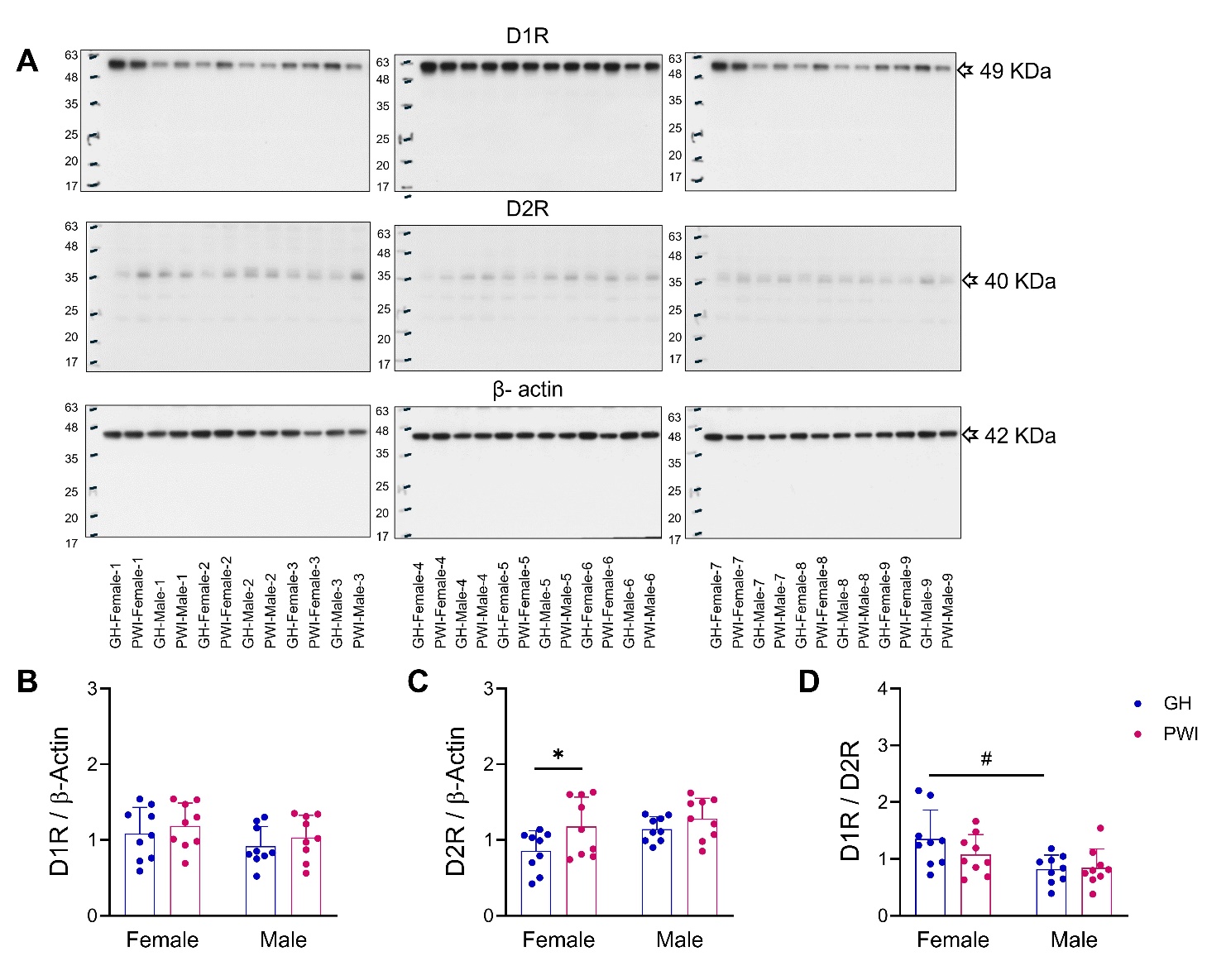


**Supplementary Figure 3****.** Levels of D1R and D2R in the mPFC of GH and PWI mice. (**A**) Images of original western blot. (**B**) Levels of D1R. (**C**) Levels of D2R. (**D**) Levels of D1R/D2R ratio. n = 9. Data were expressed as mean ± SD. Sidak’s multiple comparisons test was used following two-way ANOVA. Post-hoc results showed that ^*^*p* < 0.05 indicated significant differences between GH and PWI mice, while ^#^*p* < 0.05 represented significant differences between female and male mice.

**
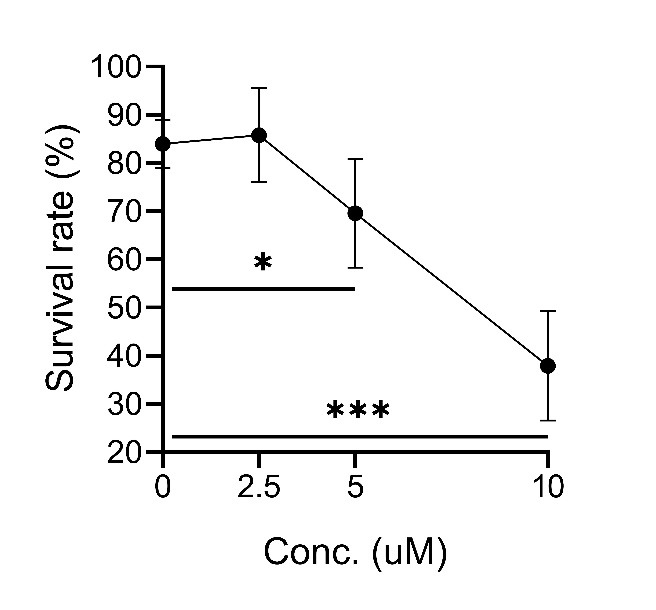
**

**Supplementary Figure 4. Cytotoxicity of SKF on BV2 microglial cells**. Data were presented as mean ± SD. n = 9. Dunnett’s multiple comparisons following the one-way ANOVAs, compared with the Veh group (0.1% DMSO in PBS). ^*^*p* < 0.05, ^***^*p* < 0.001 indicate significant differences compared to Veh treatment.


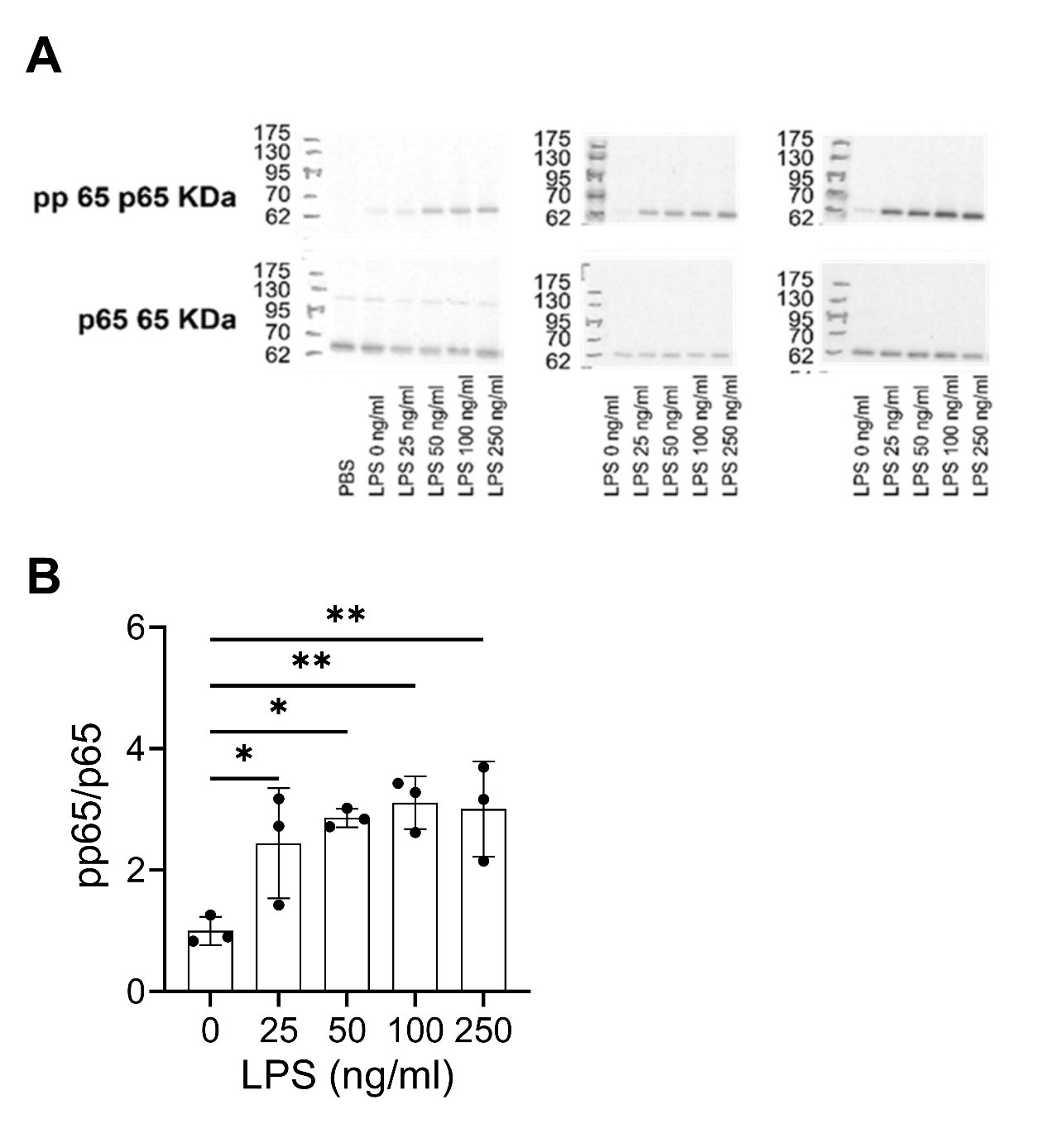


**Supplementary Figure 5. LPS dose-dependently induced p65 phosphorylation in BV2 microglial cells.** (**A**) Images of original western blot for three repeats. (**B**) Levels of pp65. Data were presented as mean ± SD. n = 3. Dunnett’s multiple comparisons following the one-way ANOVAs, compared with the 0 group. ^*^*p* < 0.05 and ^**^*p* < 0.01 indicate significant differences compared to 0 group.


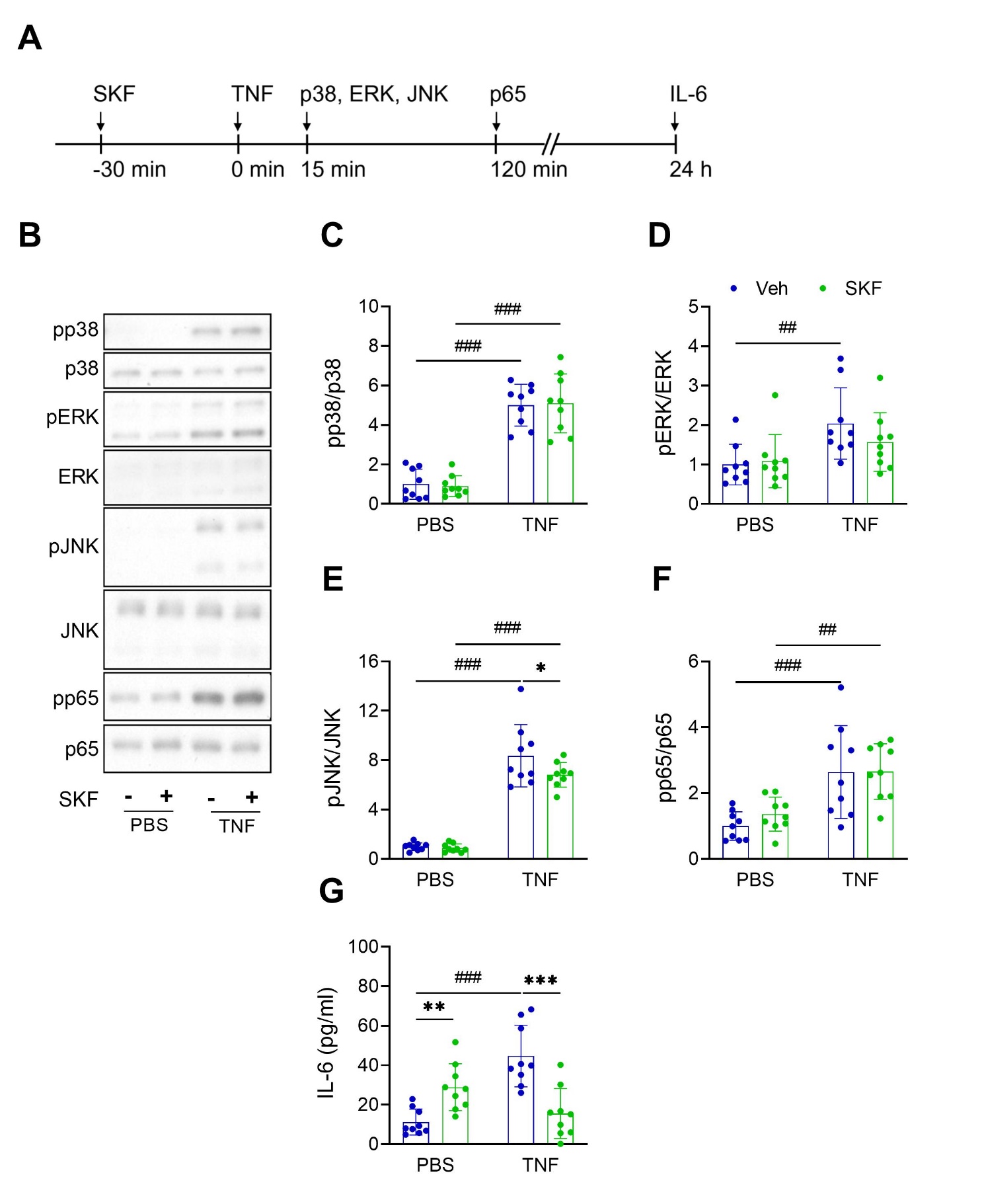


**Supplementary Figure 6. SKF partially blocked TNF-induced inflammatory responses in BV2 microglial cells**. (**A**) The experimental timeline. (**B**) Representative Western blot images. (**C**) Levels of pp38. (**D**) Levels of pERK. (**E**) Levels of pJNK. (**F**) Levels of pp65. (**G**) Levels of IL-6 in conditioned media. n = 9. Data were expressed as mean ± SD. Sidak’s multiple comparisons test was used following two-way ANOVA. Post-hoc results showed that ^*^*p* < 0.05, ^**^*p* < 0.01 and ^***^*p* < 0.001 indicated significant differences between Veh and SKF treatment, while ^##^*p* < 0.01 and ^###^*p* < 0.001 represented significant differences between PBS and TNF treatment. Please refer to **Supplementary Figure 9** for whole Western blots.

**
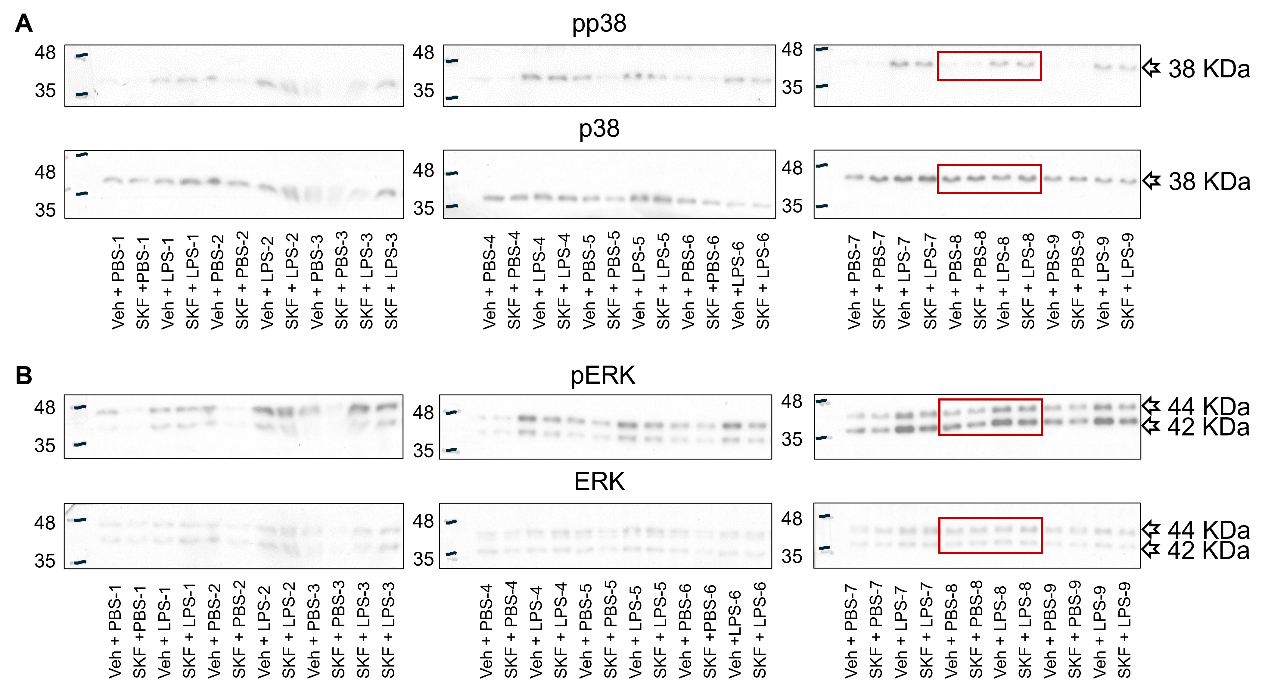

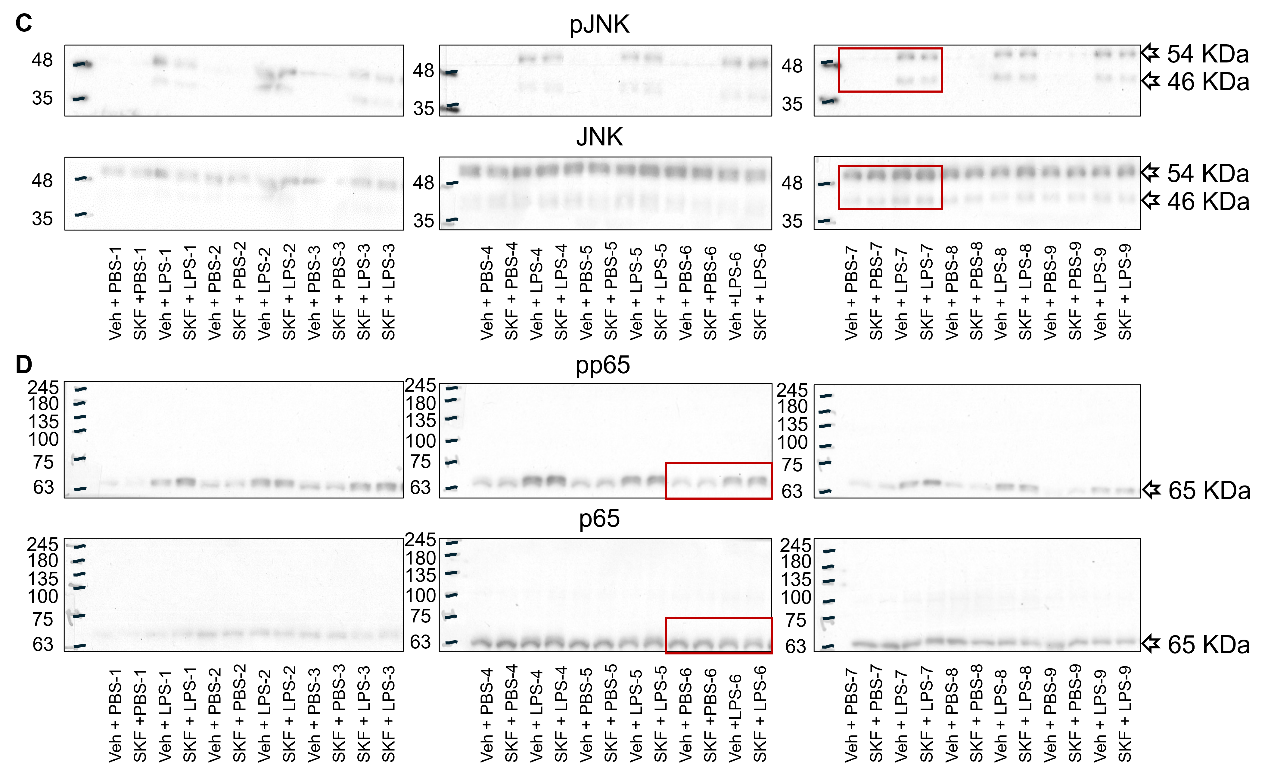
Supplementary Figure 7. Images of original western blot for three repeats of Figure 5.** The red boxed areas are presented in the Figure.


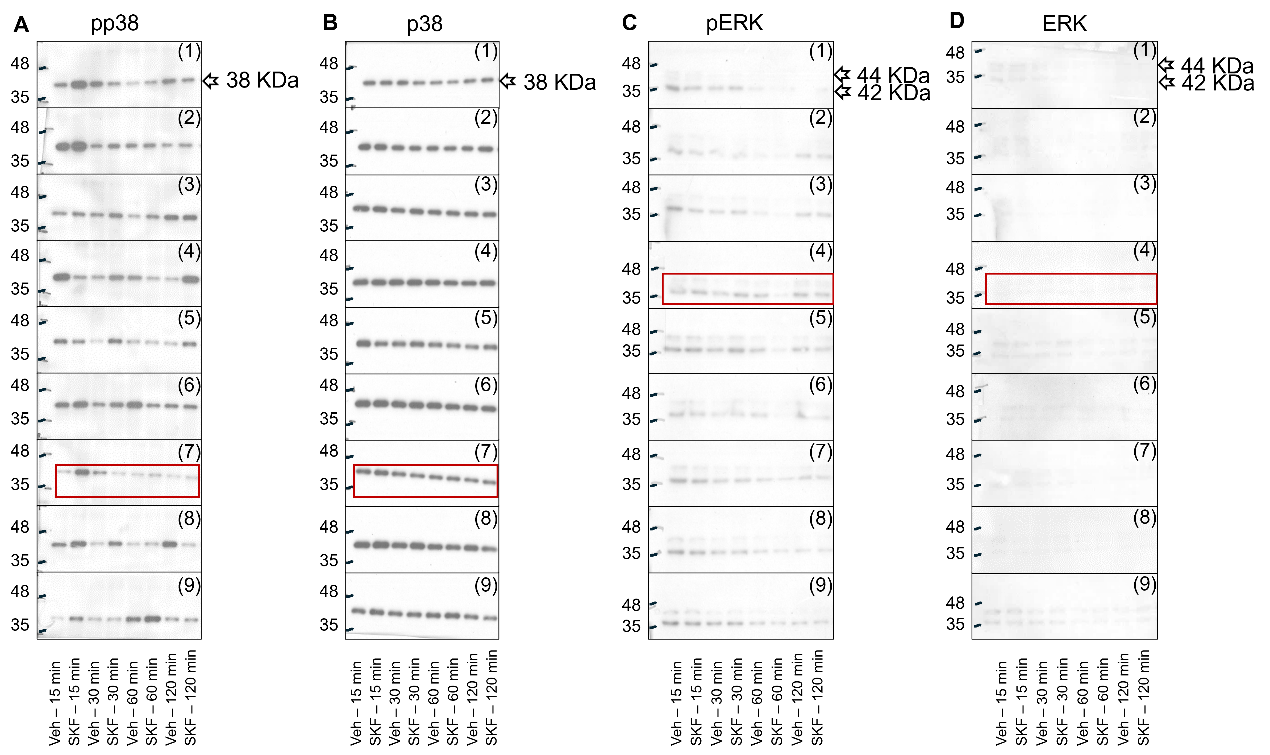


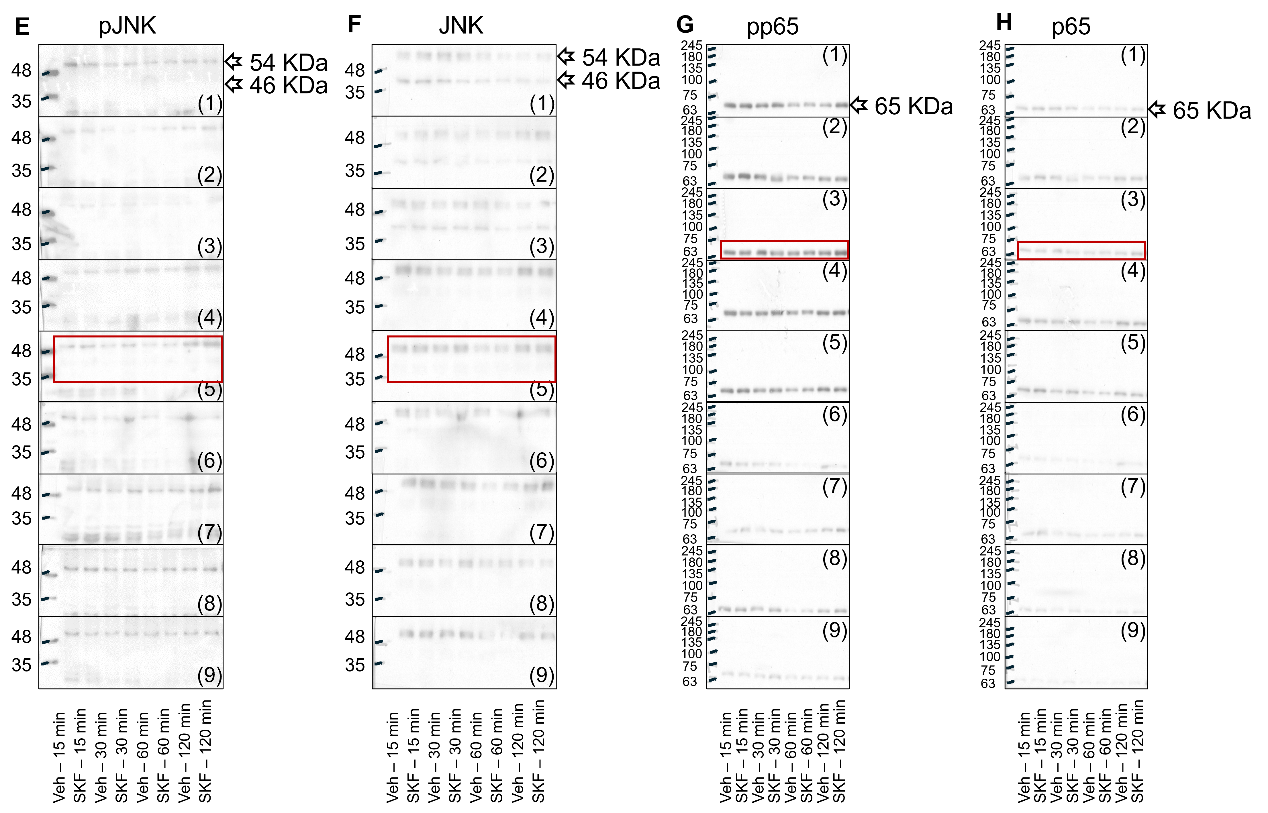
**Supplementary Figure 8.** **Images of original western blot for three repeats of Figure 6.** The red boxed areas are presented in the Figure.


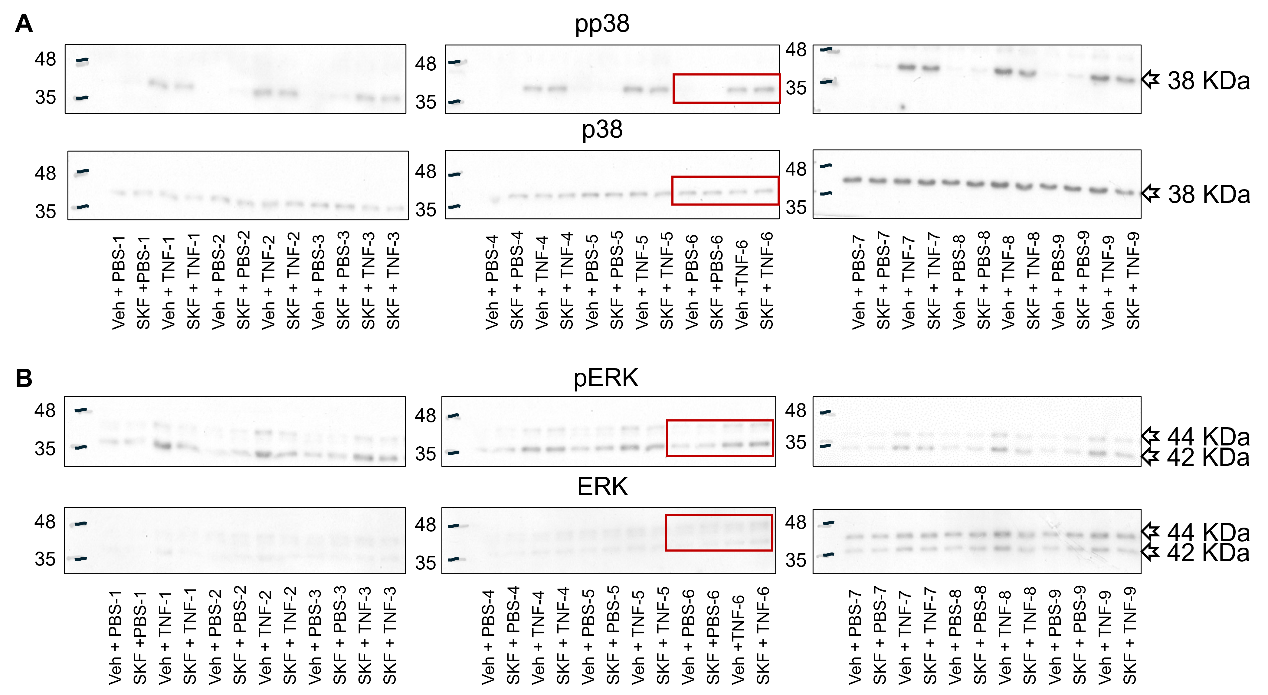

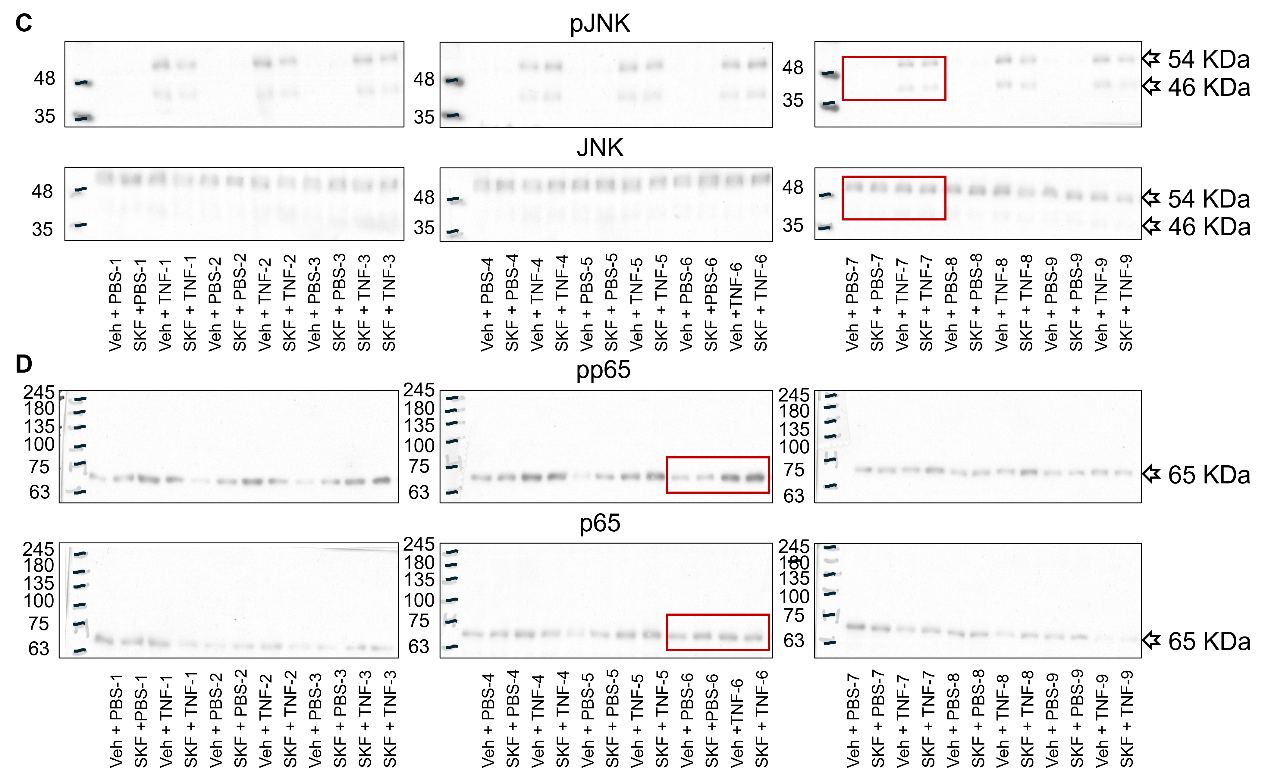
S**upplementary Figure 9. Images of original western blot for three repeats of** S**upplementary Figure 6.** The red boxed areas are presented in the Figure.
